# Identification of Conserved B and T Cell Epitopes in Glycoprotein S of Mexican Porcine Epidemic Diarrhea Virus (PEDV) Strains via Immunoinformatics Analysis, Molecular Docking, and Immunofluorescence

**DOI:** 10.3390/v18040407

**Published:** 2026-03-25

**Authors:** Jesús Zepeda-Cervantes, Alan Fernando López Hernández, Yair Hernández Gutiérrez, Gerardo Guerrero Velázquez, Diego Emiliano Gaytan Vera, Alan Juárez-Barragán, Ana Paola Pérez Hernández, Mirna G. García-Castillo, Armando Hernández García, Rosa Elena Sarmiento Silva, Alejandro Benítez Guzmán, Luis Vaca

**Affiliations:** 1 Departamento de Microbiología e Inmunología, Facultad de Medicina Veterinaria y Zootecnia, Universidad Nacional Autónoma de México, Mexico City 04510, Mexico; lopezhernandezalanfernando@gmail.com (A.F.L.H.); yairhdz2012@gmail.com (Y.H.G.); revoger@gmail.com (G.G.V.); diegomedvet135@gmail.com (D.E.G.V.); ana.pao.9928perez@gmail.com (A.P.P.H.); rosass@unam.mx (R.E.S.S.); alebenitezg@fmvz.unam.mx (A.B.G.); 2 Departamento de Química de Biomacromoléculas, Instituto de Química, Universidad Nacional Autónoma de México, Mexico City 04510, Mexico; alan.juarez.940@gmail.com (A.J.-B.); armandohg@iquimica.unam.mx (A.H.G.); 3 Facultad de Ciencias, Universidad Nacional Autónoma de México, Mexico City 04510, Mexico; mirna_garcia@ciencias.unam.mx; 4 Departamento de Biología Celular y del Desarrollo, Instituto de Fisiología Celular, Universidad Nacional Autónoma de México, Mexico City 04510, Mexico

**Keywords:** porcine epidemic diarrhea virus, Spike Glycoprotein, Major Histocompatibility Complex, peptide-binding groove, pocket, Swine Leukocyte Antigen, B cell epitope, T cell epitope, protein modeling, protein crystal, energy minimization, molecular docking analysis, immunofluorescence

## Abstract

The porcine epidemic diarrhea virus (PEDV) causes a gastrointestinal disease generating mortality rates approaching 100% in piglets worldwide. The S glycoprotein of PEDV is the main target for the development of vaccines. Two vaccines approved by the Ministry of Agriculture and Rural Development are used in Mexico: the first vaccine is based on an inactivated virus isolated more than a decade ago, whereas the second vaccine is based on mRNA technology. The most important tool for controlling PEDV outbreaks is vaccination; however, coronaviruses are characterized by the accumulation of multiple mutations, which compromise the immune response elicited by outdated vaccines. In this work, we classified the Mexican strains of PEDV reported so far in GenBank, according to their genotypes. Subsequently, we searched for B and T cell epitopes conserved in Mexican PEDV strains using bioinformatic tools. In addition, we explored whether these epitopes can induce allergies, autoimmunity, and/or toxic effects. Next, we determined the localization of B cell epitopes in the S glycoprotein using the protein crystal and protein modeling of several S glycoproteins. Finally, we carried out molecular docking analysis to assess whether these T cell epitopes could interact with the peptide-binding groove of the Swine Leukocyte Antigens (SLAs). Five conserved B cell epitopes were found to be exposed on the surface of the S glycoprotein, whereas several promiscuous CTL and HTL epitopes were bound, with low free energy, to the peptide-binding grooves of SLA-I and SLA-II, respectively. The best epitopes were used to generate a plasmid carrying the sequence to produce a recombinant protein. This plasmid was used for transfection experiments in PK-15 cell culture. The B cell epitopes reported here were recognized by the sera from pigs infected with PEDV but not by the sera from uninfected animals. These results justify future evaluations of the ability of these epitopes to stimulate cytokine production by T cells, antibody generation, and their neutralizing activity.

## 1. Introduction

The porcine epidemic diarrhea virus (PEDV) affects the intestinal tract of pigs around the world, causing watery diarrhea, vomiting, dehydration, anorexia, and fever. PEDV is especially relevant in piglets, generating mortality rates approaching 100% [1,2]. PEDV is a positive-sense, enveloped, single-stranded RNA virus (Group IV of the Baltimore classification), belonging to the order of Nidovirales from the *Coronaviridae* family of the genus Alphacoronarivus. PEDV has a diameter of 95–190 nm and a genome size of approximately 28 kb [3,4]. PEDV encodes seven open reading frames (ORFs), and four of them encode structural proteins: S glycoprotein (150–220 kDa), membrane protein (M) (20–30 kDa), nucleocapsid protein (N) (58 kDa), and envelope protein (E) (7 kDa). Two ORFs encode two non-structural polyproteins: ORF1a and ORF1b, which participate in the formation of the Replication and Transcription Complex. Finally, ORF3 encodes an ion channel involved in viral infection [3,4,5].

The S glycoprotein forms trimers and is responsible for viral entry. The S glycoprotein consists of two subunits: the S1 subunit binds to the cellular receptor, while the S2 subunit induces membrane fusion. These two subunits are the product of the processing of the S glycoprotein by transmembrane proteases. The S1 subunit has high variability and can therefore bind to a wide variety of cellular receptors, whereas the S2 subunit is more conserved [4,5].

PEDV strains are classified into two genogroups: classical strains (GI) and mutant strains (GII); GI strains were first isolated in Europe and are currently used for vaccine development, whereas GII strains contain a higher mutation rate and emerged after 2010. The GI genogroup is further divided into the subgroups GIa, GIb, and a recombinant (R) group. PEDV GIa includes strains from Belgium, China, and Korea, whereas GIb PEDV strains contain an eight-amino acid deletion in the ORF3 protein and includes strains from South Korea and China. On the other hand, subgroup R includes recombinant PEDV strains from other genogroups [6,7,8]. PEDV GII strains are further divided into INDEL S strains and Non-INDEL S strains; INDEL S strains contain deletions and insertions in the S glycoprotein, generating a lower virulence, while Non-INDEL S strains are highly virulent [5].

GII-genogroup strains (including both the INDEL S and Non-INDEL S subgroups), have been reported in Mexico since 2013. The USA/Colorado/2013 strain has been used for vaccine development, and sequence analysis has shown that it contains both conserved and variable regions. Furthermore, phylogenetic analyses have shown that this strain is distant from the strains circulating in Mexico [9,10].

One of the most effective tools to control PEDV infection is the administration of vaccines to pregnant sows. In Mexico, there are two commercial vaccines against PEDV, an inactivated vaccine from Zoetis (SAGARPA B-1196-007, Parsippany-Troy Hills, NJ, USA) and a recombinant vaccine from MSD Animal Health (SAGARPA B-0273-284, Rahway, NJ, USA) based on mRNA encapsulated into nanoparticles. At least three B cell epitopes with neutralizing capacity have been reported: COE (equivalent to CO-26K) located at residues 499–638, S1D located at residues 636–789, and 2C10 located at residues 1368–1374 [9,11]. Additionally, two non-neutralizing B cell epitopes have been identified: SS2 (748–755) and SS6 (764–771) [11]. Lactogenic immunity based on antibodies against the neutralizing epitopes of the S glycoprotein is the main protective mechanism in newborn piglets. The consumption of colostrum and milk favors the transfer of SIgA, IgG, and IgM from sows to piglets [12].

Unfortunately, the protective capacity of vaccines is compromised due to the capacity of coronaviruses to accumulate mutations; therefore, the development of vaccines against specific PEDV strains could induce a more specific immune response in vaccinated sows. As an example, the PEDV outbreak in China in 2010 had devastating effects, demonstrating the need to update inactivated and attenuated vaccines.

In this work, we classified the Mexican PEDV strains reported so far according to their genotype and obtained conserved peptide sequences from the S glycoprotein to conduct the in silico detection of B and T cell epitopes. Five out of seven B cell epitopes (S02, S03, S04, S06 and S07) were found on the surface of the S glycoprotein both in protein models and in the crystallographic structure. The most antigenic, non-allergenic, non-autoimmune and non-toxic B epitopes were used to generate a synthetic gene encoding a fusion protein containing these epitopes. The recombinant protein was expressed on the surfaces of transfected PK-15 cells as evidenced by immunostaining analysis using sera from infected pigs.

Additionally, eight helper T lymphocyte (HTL) epitopes and nine cytotoxic T lymphocytes (CTL) were predicted to bind to the peptide-binding grooves of SLA-II and SLA-I with the lowest free energy.

## 2. Materials and Methods

### 2.1. Phylogenetic Analysis of Mexican PEDV Strains

To determine the genotypes of the Mexican PEDV strains, we performed a phylogenetic analysis. The complete S glycoprotein sequences of Mexican PEDV strains were retrieved from the GenBank database, and we also compiled reference sequences from the current PEDV strains identified as G1a, G1b (INDEL), and G2. A total of 54 DNA sequences in FASTAformat were compiled and aligned using MAFFT (v. 7.526) [13]. A phylogenetic tree was constructed using the Neighbor-Joining method with the substitution model Jukes–Cantor, applying a bootstrap of 1000 resamples. The FigTree (v. 1.4.4) and Adobe Illustrator (2025) programs were used to generate the final format.

### 2.2. Detection of Conserved Peptides in Mexican PEDV Strains and Prediction of B Cell Epitopes

Peptide sequences conserved among Mexican PEDV strains were determined through MEGA11 alignment analysis [14] using the 54 S glycoprotein sequences from these strains. The conserved peptides were further analyzed to search for B cell epitopes. A B cell epitope is made up of 5–30 amino acids [15]. To look for B cell continuous epitopes of the PEDV S glycoprotein, we used the BepiPred 2.0 server [16] (by using a default selection threshold of 0.5), which uses an algorithm trained on non-epitopes and epitopes. From the resulting conserved peptides, we selected 22 peptides with the highest score.

### 2.3. Prediction of Antigenic, Allergenic, Autoimmune, and Toxic Properties of Epitopes

Predicted B cell epitopes were analyzed using the following servers: VaxiJen v2.0 (threshold above 0.5) [17], AllerTOP v2.1 [18], BLASTp (v. 2.17.0) (E value of 0.05) [19], and ToxinPred (v. 2) (threshold above 0.00, SVM-based method (Swiss-Prot)) [20], to predict their antigenic, allergenic, autoimmune, and toxic properties, respectively. Those epitopes that proved to be good candidates were analyzed with VaxiJen v2.0 to predict their antigenicity with a threshold of 0.5 selecting the organism “virus” and their allergenicity with AllerTOP v2.0. The risk of autoimmunity was tested with a Blastp analysis selecting the species *Sus scrofa* and *Sus scrofa domestica* as the target. The prediction of T cell epitopes was performed with servers servers based on chromatography experiments and training algorithms [21].

### 2.4. Epitope Localization Using a Highly Homologous S Glycoprotein Crystal

The next step was to visualize the location of the PEDV epitopes. To do this, we used two strategies: for the first strategy we used the crystal structure with the highest percentage of compatibility with a Mexican PEDV strain donated to our laboratory (EdoMex/205/2018 accession no. MT490316.1) and the highest percentage of resolved amino acids suggested based on the RCSB Protein Data Bank (RCSB PDB). A crystal of the glycoprotein S with high homology obtained from the PDB (PDB 7W6M) was used as a model. The predicted B cell epitopes located on the surface were marked using Chimera X (v. 1.8) software. Due to the lack of crystals for the S glycoprotein from Mexican PEDV strains, we modeled and refined the S glycoprotein from Mexican PEDV strains. From the 54 complete sequences of the glycoprotein S present in the GenBank database, one PEDV strain was randomly selected by year to visualize the B cell epitopes: Mich(2013)MH006965.1, Tlax(2014)MN091346.1, Ver(2015)MH013464.1, Pue(2016)MH006963.1 Jal(2017)MH004420.1, and EdoMex(2018)MT490316.1. The amino acid sequences of the glycoprotein S were used to generate protein models, using the AlphaFold 3 server [22]. The modeled PEDV S glycoproteins were submitted to the PROCHECK server [23] to verify the protein and select the best candidates. Finally, the modeled PEDV S glycoproteins were subjected to energy minimization by using the YASARA server [24].

### 2.5. Reagents, Cells, and Plasmids

Dulbecco’s Modified Eagle Medium (DMEM) (Cat. No. 292805), Optimem™ (Cat. No. 22600-043), Hoechst 33342 (Cat. No. 62249), and FITC-Protein A (Cat. No. 10–1011) were purchased from Thermo Fisher Scientific (Waltham, MA, USA). Phosphate-buffered saline (PBS) 1× was prepared according to laboratory standards (pH 7.2, 137 mM NaCl, 2.7 mM KCl, 10 mM Na2HPO4, 1.8 mM KH2PO4), and heat-inactivated Bovine Serum Albumin (BSA) (Cat. No. A3311-50G) was from Sigma-Aldrich (Burlington, MA , USA).

PK-15 (porcine kidney) cells (ATCC^®^ PTA-8244™) were obtained from the American Type Culture Collection (ATCC, Manassas, VA, USA) and maintained in DMEM supplemented with 10% heat-inactivated fetal bovine serum (FBS) (Cod. 16629525 from Gibco, Waltham, MA, USA) (56 °C for 30 min).

The recombinant plasmid PEDVV5 (GeneArt™, ThermoFisher Scientific, Waltham, MA, USA) was developed, including the gene encoding a signal peptide, the B cell epitopes of the S glycoprotein of PEDV separated by linkers, a transmembrane domain, the PADRE epitope, c-Myc, and 6Xhis. This gene was flanked by two restriction sites, BmtI (BspOI) at the 5′ end and Bsp1407I (BsrGI) at the 3′ end, and inserted into the multicloning site under the cytomegalovirus (CMV) promoter using the pCDNA3.1 plasmid. This plasmid was amplified in competent TOP10 cells (ThermoFisher Scientific, Waltham, MA, USA) and purified using the QIAGEN Plasmid Midi kit (cat. no. 12143, QIAGEN, Hilden, Germany).

### 2.6. Cell Transfection and Immunofluorescence Assay

PK-15 cells were cultured at a density of 2 × 10^4^ cells in 96-well microplates. The cells were transfected with the plasmid PEDVV5. Following transfection, cells were processed for immunofluorescence against PEDV, utilizing non-transfected cells as negative controls.

Cells were washed twice with PBS and incubated with Hoechst 33342 (1:500 dilution) for 5 min to label the nucleus. Subsequently, cells were fixed with 4% paraformaldehyde for 20 min and permeabilized with PBS containing 0.3% Triton X-100 for 15 min. To quench residual aldehydes, cells were treated with 50 mM glycine in PBS for 10 min. Blocking was performed using 5% BSA in PBS for 1 h at room temperature. The cells were then incubated with anti-PEDV serum obtained from infected pigs (diluted 1:500 in PBS containing 2% BSA) for 1 h at room temperature. To visualize antibody binding, FITC-conjugated Protein A was added at a dilution of 1:5000 and incubated for 1 h at room temperature. Microplates were washed three times with PBS after each incubation step. Images were acquired using an epifluorescence microscopy (OLYMPUS IX71).

### 2.7. Quantification of Immunofluorescence in PK-15 Cells

The fluorescence intensity of PK-15 cells transfected with the plasmid PEDVV5 was analyzed using ImageJ (v. 1.54g). Briefly, circular regions of interest (ROIs) were drawn around each cell, and the total fluorescence intensity was measured for each cell from two independent immunofluorescence experiments. 

### 2.8. Prediction of Helper T Cell Epitopes

To search for HTL epitopes, we used the NetMHCIIpan-4.1 server searching for peptides with a length of 11–15 residues as previously reported [24]. The additional configuration was as follows: threshold for strong binder ligands less than 1%, and for weak ligands, it was less than 5%. Since the option of using SLA-II is not available on this server, we selected the HLA alleles DRB1*1101, DRB1*0401, and DRB1*1301, which have been shown to be useful for predicting HTLs in other cloven-hoofed mammals [25]. The safety of HTL epitopes was analyzed with the same servers mentioned in point 4.3 of this work.

### 2.9. Prediction of CTL Epitopes

The server NetMHCpan-4.1b [26] was used to predict CTL epitopes ranging from eight to nine residues. The SLA-1*0401, SLA-2*0401, and SLA-3*0401 alleles, belonging to the Major Histocompatibility Complex Type I (MHC I), were selected because they are constitutively expressed in the nucleated cells of pigs of several representative breeds [27]. The selection range for strong ligands was less than 0.5%, and for weak ligands, it was less than 2%. The safety of CTL epitopes was analyzed as reported above.

### 2.10. Molecular Docking Analysis

A molecular docking analysis was performed to determine whether the predicted T cell epitopes can bind the MHC. For this, T cell epitopes that met all criteria were modeled with AlphaFold 3.0 and analyzed using UCSF Chimera 1.18, where charges and hydrogen bonds were added, and the structures were minimized to save them as molecular docking ligands in PDB format. For the molecular docking of CTL epitopes with MHC-I, we used an SLA-I molecular structure from a co-crystallization experiment containing SLA-1*0401 and an Ebola virus peptide (ID: 3QQ4) obtained from the PDB. The structure of SLA-1*0401 was separated from the Ebola virus peptide using UCSF Chimera 1.18. The rest of SLA-I (SLA-2:0401 and SLA-3:0401 with ID numbers: SLA06148 and SLA06198, respectively) and SLA-II sequences (SLA-DRB1:0401, SLA-DRB1:1101, and SLA-DRB1:1301, with ID numbers: SLA06016, SLA06052, and SLA06058, respectively) were obtained from the Immuno Polymorphism Database (IPD) (https://www.ebi.ac.uk/ipd/mhc/, accessed on 3 July 2025). These SLA molecules were subsequently modeled with the AlphaFold server (v3) [28] and refined with the GalaxyRefineComplex server [29] (https://galaxy.seoklab.org/cgi-bin/submit.cgi?type=COMPLEX, accessed on 4 July 2025). The best model for each SLA was selected with Procheck [22] (https://saves.mbi.ucla.edu/, accessed on 6 July 2025) and ProSA [30] (https://prosa.services.came.sbg.ac.at/prosa.php, accessed on 3 February 2026) to obtain the Ramachandran plot and the Z-score, respectively. HTL epitopes were tested as ligands of different SLA-II molecules (SLA-DRB1:1101, SLA-DRB1:0401, and SLA-DRB1:1301). Protein–peptide molecular docking was performed using the HADDOCK 2.4 server [31] (https://rascar.science.uu.nl/haddock2.4/, accessed on 3 February 2026), and the binding energy prediction was calculated using the PROtein binDIng enerGY prediction (PRODIGY) server [32] (https://rascar.science.uu.nl/prodigy/, accessed on 3 February 2026). Finally, the results were visualized using PyMOL (v 3.1.7.2), and the free energy resulting from the interaction of epitopes and SLA was reported.

## 3. Results

### 3.1. Mexican Variants of PEDV Belong to the Genotype G1b INDEL and G2

**Figure 1:**
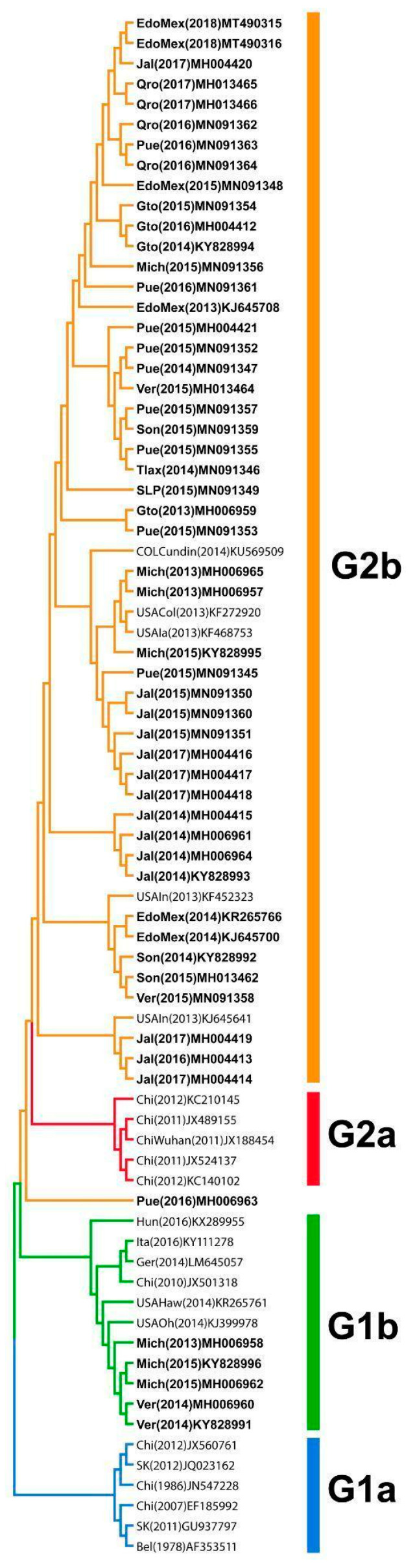
Neighbor-Joining phylogenetic tree of 54 Mexican PEDV strains. The tree indicates four main groups: G1a (blue), G1b INDEL (green), G2a (red), and G2b (orange). Mexican strains are highlighted in bold.

For the development of immunogen-based strategies against coronavirus, it is important to know the PEDV genogroups circulating in Mexico. To this end, we analyzed the complete nucleotide sequences of the S glycoprotein from all the Mexican PEDV strains reported in GenBank, as well as of the representative PEDV strains for G1a, G1b, G2a, and G2b reported in other countries. A total of 54 Mexican PEDV strains were identified (Table S1) and compared with the vaccine strain based on a strain from Colorado, USA, as well as strains from Colombia, Europe, and Asia (Table S2). The phylogenetic tree revealed the grouping of the Mexican strains into genogroups G1b (INDEL) and G2b (Figure 1).

### 3.2. Conserved Epitopes Among Mexican PEDV Strains

The next step was to identify conserved epitopes in the S glycoprotein from Mexican PEDV strains. Briefly, amino acid alignment analysis was performed to determine the conserved peptides in the S glycoprotein of PEDV from Mexican strains (Table S3). These peptides were then used to predict B cell epitopes using BepiPred 2.0 software. Twenty-two peptides with the highest antigenicity were selected and proposed as potential B cell epitopes during the first selection with the BepiPred 2.0 server (Table S4).

### 3.3. Seven Epitopes Conserved in the S Glycoprotein of Mexican PEDV Strains Are Safe and Antigenic

For the selection of B cell epitopes from the PEDV S glycoprotein, a second filter was used with the VaxiJen v2.0 server. The 22 potential epitopes identified from the first detection were subjected to a further analysis to identify the possible antigenic, allergenic, autoimmune, and toxic effects. The preliminary results indicated that only four epitopes met all the requirements (Table S5). In addition, we found three other epitopes that met almost all the criteria but were potentially allergenic; therefore, a subsequent Protein Blast analysis was performed, which indicated that the allergenic sequences did not match these PEDV epitopes (Table S6). Seven conserved sequences from the PEDV S glycoprotein that met all the criteria were proposed as potential B cell epitopes.

### 3.4. Five Conserved B Cell Epitopes Are Located on the Surface of the PEDV S Glycoprotein

Next, we conducted an in silico analysis to identify the location of epitopes in the S glycoprotein using the crystal structure reported for the PEDV S glycoprotein. Because crystal structures for the S glycoproteins from Mexican PEDV strains have not been reported, we conducted energy minimization to improve the quality of molecular modeling of the S glycoproteins (Figure S1 to visualize the PROCHEK analysis).

**Figure 2:**
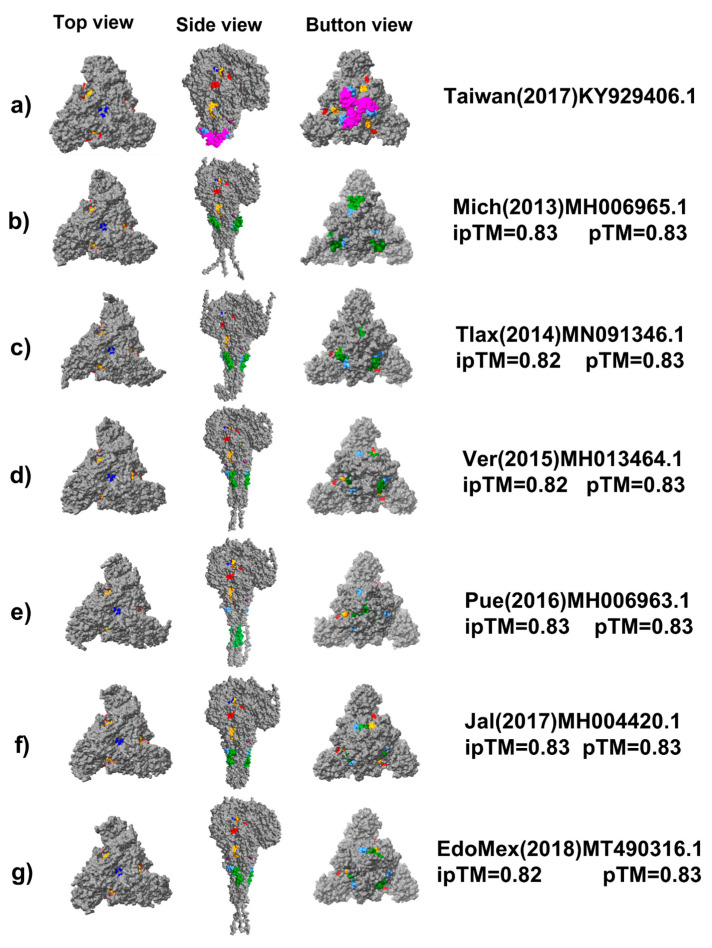
Location of B cell epitopes in a crystal structure of the PEDV S glycoprotein with high homology (PDB 7W6M) and in the modeling of the S glycoprotein from Mexican PEDV strains. (**a**) Visualization of B cell epitopes of the S glycoproteins from Mexican PEDV strains in a protein crystal with high homology (KY929406.1). (**b**) Modeling of the S glycoprotein of PEDV isolated in Michoacan (GenBank access number: MH006965.1), (**c**) Tlaxcala (GenBank access number: MN091346.1), (**d**) Veracruz (GenBank access number: MH013464.1), (**e**) Puebla (GenBank access number: MH006963.1), (**f**) Jalisco (GenBank access number: MH004420.1), and (**g**) Estado de Mexico (GenBank access number: MT490316.1). The conserved B cell epitopes were visualized to know their location on the surface of the PEDV S glycoprotein using Chimera X software. From left to the right: top view, side view, and bottom view. Colors indicate the different epitopes found: epitope S01 and epitope S05 are located within a pore of the glycoprotein and are represented in blue (hidden), epitope S02 is highlighted in red, epitope S03 is highlighted in yellow, epitope S04 is highlighted in purple, the epitope S06 is highlighted in sky blue, and epitope S07 is highlighted in green. The area highlighted in magenta corresponds to an amino acid sequence close to epitope S07, which is absent in the crystal (not resolved). * ipTM: interface-pTM; pTM: Predicted Template Modeling. Obtained from structural analysis with AlphaFold server (v3).

S glycoprotein models from Mexican PEDV strains were highly compatible with the reported crystal. Four exposed epitopes were found in the crystal structure of the S glycoprotein, because the fifth epitope is unresolved in the crystal structure (Figure 2 and Table 1), while the five exposed epitopes were found on the surface of the S glycoprotein models in all Mexican PEDV strains analyzed. Thus, the structural analysis obtained with the S glycoprotein crystal and S glycoprotein models were very compatible.

### 3.5. B Cell Epitopes Are Recognized by Sera from PEDV-Infected Pigs in Transfected PK-15 Cells

**Figure 3:**
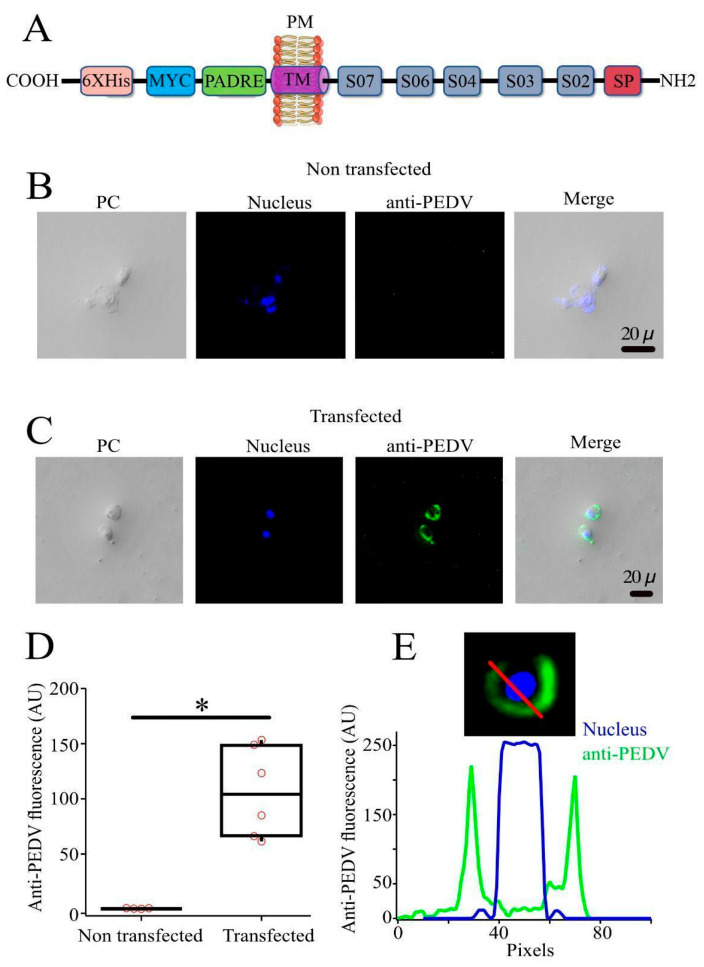
Immunofluorescence analysis of PK-15 cells transfected with the recombinant plasmid PEDVV5. (**A**) The recombinant protein PEDVV5 was constructed containing a signal peptide (SP) followed by the conserved B cell epitopes S02, S03, S04, S06, and S07 that were placed at the N-terminus, while the transmembrane domain and the epitopes PADRE, c-Myc, and polihistidine (6Xhis) were placed at the C-terminus. (**B**) Non-transfected PK-15 cells were treated via specific immunofluorescence against PEDV. (**C**) PK-15 cells transfected with the plasmid PEDVV5 were processed via PEDV-specific immunofluorescence using anti-PEDV sera from infected pigs and FITC-conjugated protein A; the nuclei were stained with Hoechst. All microscopy images are of the same scale (20 μm). PC indicates phase contrast. (**D**) Quantification of the anti-PEDV fluorescence intensity expressed in arbitrary units (AU) for non-transfected and transfected cells. Each symbol represents the total fluorescence intensity measured from a single cell. All cells are plotted using a box plot showing the lower limit (25th percentile), center (50th percentile), and upper limit (95th percentile) of fluorescence intensity. Statistical analysis was performed using a Student’s t-test to compare the means of the two groups shown. This test is appropriate for small sample sizes, such as the number of cells illustrated in Figure 3B–D. Statistically significant differences are indicated by * (*p* < 0.01). (**E**) Fluorescence intensity profile along a transfected cell (indicated with a red line in the selected image) showing the distribution of the anti-PEDV signal (green) and the Hoechst-stained nucleus (blue). Plot line analysis of the fluorescence intensity in a single cell transfected with the recombinant plasmid PEDVV5. The upper panel shows the fluorescence intensity from the nucleus (Hoechst, blue) and PEDV signal (green). The red line was used to measure the fluorescence intensity. Measurements were conducted using the Plot line plugin from ImageJ.

An efficient vaccine most activate B cells by inducing the crosslinking of their B cell receptors (BCRs). To achieve this, antigens must be exposed on the cell surface as multimeric antigens. Therefore, we developed a recombinant protein that mimics the short structure of the PEDV S glycoprotein and promotes its expression on the surfaces of transfected cells. To achieve surface expression, the construct contained a transmembrane domain. The recombinant plasmid PEDVV5 was designed to encode a recombinant protein containing the following elements: a signal peptide and B cell epitopes at the N-terminus, followed by a transmembrane domain, the PADRE epitope, c-Myc, and 6Xhis at the C-terminus (Figure 3A). The recombinant protein was expressed in PK-15 cells and identified via immunofluorescence. PK-15-transfected cells showed strong labeling by sera from infected pigs after 48 h of transfection (Figure 3B,C), whereas non-transfected cells did not show sera labeling (Figure 3C,D). A circular pattern around each cell was observed, strongly suggesting labeling at the plasma membrane (Figure 3E).

### 3.6. Eight Conserved Peptides Can Bind to MHCII

The T helper cellular immune response is important to induce the production of antibodies and cytotoxic responses. Therefore, we searched for helper T cell epitopes of 11–15 amino acid residues in the conserved peptide sequences in the PEDV S glycoprotein using the NetMHCIIpan 4.1b server. Preliminary results showed 52 weak binders and 2 strong binders (Tables S7–S12). According to this server, the eluted ligand range must be <1% to be considered a strong ligand, while <5% is considered a weak ligand. The higher binding affinity range indicates possible binding to MHC-II. Because the NetMHCIIpan server does not allow the selection of any type of SLA-II, in this first screening, we used the HLA-II options as reported elsewhere [25]. The epitopes HTL16 (VVTYVNLTRDQLPD) and HTL36 (CVVTYVNLTRDQLPD) showed a stronger interaction with HLA-II DRB1*1101, whereas the rest of the epitopes are predicted as weak binders. Additionally, this analysis demonstrated that the YKRCSNGRS core is shared by 10 epitopes and interacts with both HLA-II DRB1*1101 and HLA-II DRB1*0401. Further analysis was performed to assess the safety of the 54 helper T cell epitopes from which 8 safe conserved epitopes were identified (Table 2, Tables S13 and S14).

### 3.7. Conserved HTL Epitopes Interact with the SLA-II

**Figure 4:**
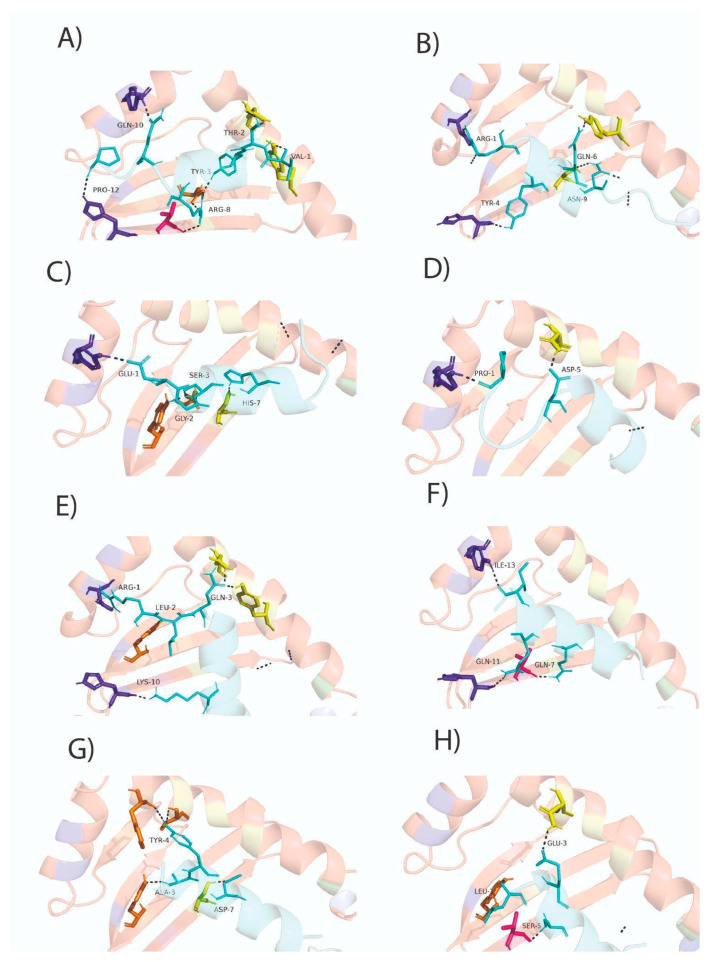
Molecular docking shows the binding of HTL epitopes derived from PEDV S glycoprotein to the pockets of the peptide-binding groove of SLA-II (SLA-DRB1*1101). (**A**) HTL1 interacts through four pockets (P4, P6, P7, and P9); (**B**) HTL09 interacts with two pockets (P4 and P9); (**C**) HTL12 interacts with three pockets (P4, P7, and P9); (**D**) HTL13 interacts with two pockets (P4 and P9); (**E**) HTL17 interacts with three pockets (P4, P6, and P7); (**F**) HTL27 interacts with two pockets (P6 and P9); (**G**) HTL41 interacts with two pockets (P4 and P7); and (**H**) HTL49 interacts with three pockets (P4, P6, and P7) of the SLA-II beta chain. The HTL epitopes are observed in cyan, whereas the pockets belonging to the beta chain, P1, P4, P6, P7, and P9, are observed as sticks in green, yellow, hot pink, orange, and purple blue, respectively.

HTL responses help B and T cells perform their effector activities through cytokines, receptors, and signaling pathways, promoting the production of various antibody isotypes and cytotoxic activity. To determine whether the eight HTL epitopes present in the S glycoprotein could be coupled to the antigen-binding groove of SLA-II molecules, a molecular docking analysis was conducted. Because no crystal structure is reported for SLA-II, we conducted modeling of SLA-II molecules, as well as energy minimization (Figure S2). Molecular docking analysis revealed that the eight HTL epitopes analyzed may bind to the beta chain of SLA-II (SLA-DRB1*1101) occupying 2–4 pockets (Figure 4). HTL01 binds to four major pockets (P4, P6, P7 and P9), HTL12, HTL17, and HTL49 bind to three pockets (P4, P7, and P9 for HTL12; and P4, P6, and P7 for HTL17 and HTL49), and HTL09, HTL13, HTL27, and HTL41 bind to two pockets (P4 and P9 being the most frequently used) (Figure 4). These eight epitopes were predicted to bind to SLA-DRB1:0401, SLA-DRB1:1101, and SLA-DRB1:1301 with the lowest free energy (Table 3).

### 3.8. Spike Glycoprotein Contains Nine Peptides That Bind to SLA-I

**Figure 5:**
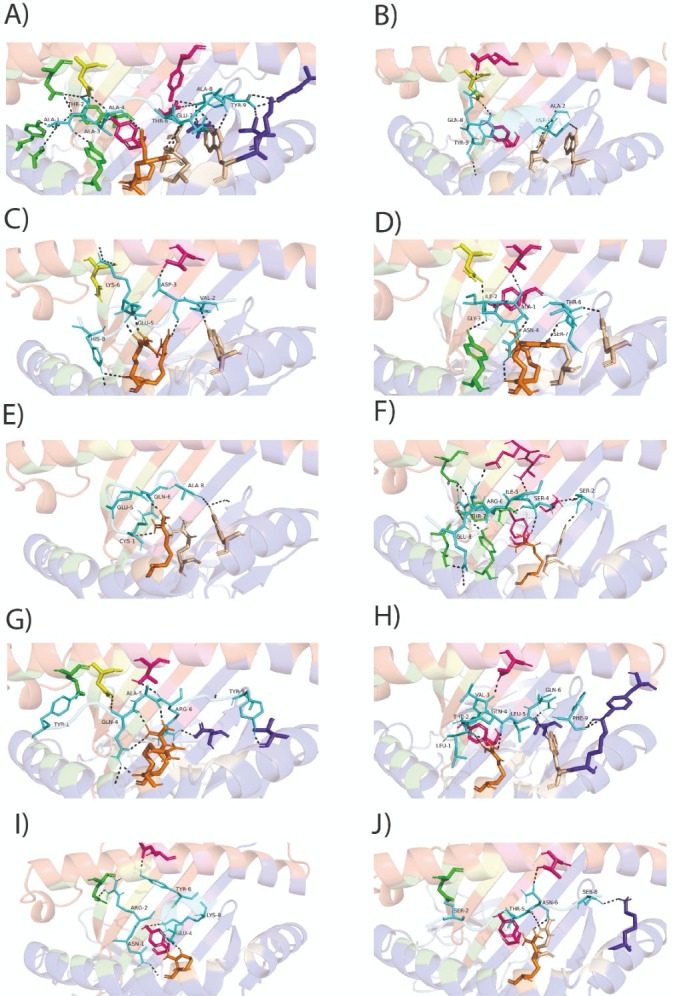
Molecular docking of the predicted CTL epitopes and SLA-I (SLA-I*0401). (**A**) Molecular docking analysis between the AY9 peptide (ATAAATEAY) derived from the Ebola virus VP35 and SLA-I*0401 obtained by performing a co-crystallization experiment (PDB accession number: 3QQ4). The interaction of the AY9 peptide with the six SLA-I pockets (pockets (**A**–**F**)) is observed, as in the original experiment [33]. (**B**) CTL04 interacts with three pockets (**B**,**C**,**E**); (**C**) CTL05 interacts with four pockets (**B**–**E**); (**D**) CTL06 interacts with five pockets (**A**–**E**); (**E**) CTL12 interacts with two pockets (**D**,**E**); (**F**) CTL03 interacts with four pockets (**A**,**C**–**E**); (**G**) CTL17 interacts with five pockets (**A**–**D**,**F**); (**H**) CTL18 interacts with four pockets (**C**–**F**); (**I**) CTL20 interacts with three pockets (**A**,**C**,**D**); and (**J**) CTL22 interacts with five pockets (**A**,**C**–**F**) of the SLA-I. The PEDV S glycoprotein-derived CTL epitopes are represented in cyan, and the six pockets (**A**–**F**) of the SLA-I heavy chain are represented by green, yellow, hot pink, orange, wheat, and purple blue sticks, respectively.

CTL is responsible for lysing cells infected with intracellular pathogens. CTL epitopes were predicted using several tools. In the first screening, we used the NetMHCpan 4.1 server. Peptides of eight amino acid residues showed weak binding, whereas peptides of nine amino acid residues exhibited stronger binding (Tables S15 and S16). Thus, only strong binders of nine amino acid residues were used to predict CTL epitopes conserved in the PEDV S glycoprotein. Our analysis revealed that 25 potential CTL epitopes are present in the PEDV S glycoprotein, which may bind to SLA-1:0401, SLA-2:0401, and/or SLA-3:0401. Six of them may be promiscuous CTL epitopes, which may interact with several SLA-Is (Table S17). Of these predicted 25 CTL epitopes, only 6 met all criteria for low antigenicity, non-allergenic, non-autoimmune, and not toxic effects. Three epitopes were predicted to be moderately allergenic. Further analysis demonstrated that these three CTL epitopes did not significantly resemble their allergenic counterparts (Table 4 and Table S18). Additionally, our molecular docking analysis showed that all nine CTL epitopes were coupled to SLA-I*0401 (Figure 5) with the lowest free energy. Similar results were obtained when modeling with SLA-2*0401 and SLA-3*0401 (Figure S3 and Table 5).

## 4. Discussion

PEDV is a highly contagious coronavirus affecting pigs. Pig infection leads to severe enteric disease characterized by diarrhea, vomiting, and high mortality rates, especially in newborn piglets. This virus is distributed around the world and is transmitted through fecal–oral routes [34]. Infected animals can shed the virus in their feces, facilitating rapid transmission within herds [2]. Since its emergence in America in 2013, PEDV has been a cause of great concern in the swine industry due to its economic impact and the challenge of controlling its dissemination [2]. During the replication of coronavirus in the host, many mutations are generated resulting in novel strains. Furthermore, recombination between coronaviruses has also been reported, which is associated with the presence of strains with insertions/deletions (INDELs), primarily located in the S glycoprotein. Changes in the S glycoprotein sequence are related to changes in the virulence of these strains, affecting the effectiveness of current vaccines [35,36]. In addition, the evolution of RNA viruses is driven by its mutation rate, which is higher than DNA viruses [37].

During the pathogenesis of PEDV, this virus enters enterocytes through the interaction of the viral S glycoprotein with cellular receptors. Unlike other coronaviruses, the S glycoprotein of PEDV is not cleaved into S1 and S2 segments, although for practical purposes, it has been divided into S1 and S2 as with other coronaviruses. The S1 region (1–789) is the region most accessible to antibodies, containing a spherical head, while the S2 region (790–1383) forms the stem portion and is poorly immunogenic [38]. Overall, antibodies against the S glycoprotein can neutralize infection, avoiding entry and/or fusion to the host cell membrane. Vaccination has become a cornerstone strategy to mitigate the effects of this disease [39]. The main strategy to control PEDV outbreaks is to generate lactogenic immunity through sow vaccination. Current vaccines have shown promise in reducing the severity of clinical signs and mortality in infected piglets, helping to protect the health and welfare of pigs [39].

Currently, inactivated and recombinant vaccines are marketed in the United States, whereas in Asia, there is a great diversity of inactivated vaccines based on different strains, which aid in reducing the morbidity and mortality [12]. In Mexico, there are two PEDV vaccines: one of them is based on an inactivated virus containing a PEDV strain isolated from Colorado, USA in 2013. The second vaccine is based on mRNA technology. It is relevant to mention that there are no current vaccines targeting the strains circulating in Mexico. An attractive alternative to traditional vaccines is the development of multi-epitope vaccines based on the local circulating strains, which offers many advantages including that they can cover all the local circulating PEDV strains identified in the country. In addition, unlike vaccines generated with inactivated viruses, recombinant vaccines cannot revert to virulent phenotypes or recombine with other PEDV strains. They are also safer as they do not contain toxic, allergenic or autoimmune epitopes (unlike vaccines based on inactivated virus).

Although there are some commercial vaccines and recombinant vaccines under development in several continents, the high genetic and antigenic variability of PEDV may compromise the efficacy of such novel vaccines.

In this regard, the generation of a vaccine based on conserved epitopes found in the S glycoprotein may facilitate vaccination without having to generate new vaccines for emerging circulating strains [15,39].

In an attempt to postulate epitopes that may serve in the future as the backbone of new vaccines, we first classify the PEDV strains circulating in Mexico [10]. We performed a phylogenetic analysis, which revealed that the Mexican PEDV strains belong to the G2b and G1b genotypes. These strains were not related to strains of the G1a and G2a genotypes. PEDV strains isolated in Mexico belong to the G2 genotype, whereas a few PEDV strains from Michoacan and Veracruz belong to the G1b genotype. These results are consistent with those obtained by Reveles-Felix (2019), where only a few Mexican PEDV strains were classified as G1b (including some from Michoacan and Veracruz states) [10].

For the development of multi-epitope vaccines, it is necessary to identify conserved regions that may be immunogenic and provide coverage for most of the circulating PEDV strains. Particularly, in the PEDV S glycoprotein, there are some antigenic peptides that have been reported in the literature, such as COE, S1D, and 2C10. One of the first PEDV peptides was originally found in the N-terminal region of the TGEV (Transmissible Gastroenteritis Virus) S glycoprotein, spanning amino acid residues 503–715 (CO-26K). CO-26K shares more than 90% homology with Feline Infectious Peritonitis Virus (FIPV) and only 28% with PEDV. The CO-26K equivalent (COE) was found in the PEDV S glycoprotein (499–638 amino acids), and it has been shown to induce the production of neutralizing antibodies against PEDV [40,41,42,43].

Another antigenic peptide identified is S1D (636–789). The fusion of S1D with cholera toxin B subunit elicited mucosal and systemic immune responses generating IgA and IgG, respectively [38,44]. In turn, two short peptides were identified in the S1D region: S1D5 (744–759) and S1D6 (756–771), which contain two core epitopes: SS2 (748–755) and SS6 (764–771). Although antibodies against S1D5 and S1D6 were able to recognize the S glycoprotein of PEDV, however, the generated antibodies had no neutralizing activity in cell cultures [38].

2C10 is another antigenic peptide from the PEDV S glycoprotein with neutralizing activity, originally identified via phage display. The peptides SHRLP(Y/Q)(P/V) and GPRPVTH present in phages resemble the GPRLQPY peptide (1368–1374) located in the cytoplasmic domain of the S glycoprotein. Since the location of this peptide cannot explain the generation of neutralizing antibodies, it was suggested that 2C10 might be part of a conformational epitope [45]. Although some of these PEDV S glycoprotein-derived peptides produced neutralizing antibodies, they are poorly conserved among PEDV strains (Figure S4).

We found that the COE and S1D peptides differ in some amino acids in the Mexican PEDV strains (Figure S4A,B). The shortest neutralizing peptide 2C10 found in S2 (1368–1374 amino acid residues) is also not conserved in Mexican PEDV strains (Figure S4C). Additionally, we have found that even epitopes that do not generate neutralizing antibodies such as SS2 and SS6 are not entirely conserved in Mexican strains (Figure S4D). These results are consistent with those reported by Cruz et al. (2006) [45], where COE was not conserved among Korean PEDV strains. Since the conserved peptides reported with neutralizing activity are not fully conserved in Mexican PEDV strains, it was important to search for new candidates for conserved epitopes in all Mexican PEDV strains. Our protein alignment analysis yielded 22 conserved peptides. From these 22 peptides, we performed a second screening to predict their immunogenic capacity, in addition to their capacity to be allergenic, autoimmune, or toxic. From this second round of analysis, we identified seven B cell epitopes, of which four were apparently exposed on a PEDV S glycoprotein crystal (one epitope was not resolved in this structure). An additional B cell epitope was identified in the modeled S glycoproteins from Mexican PEDV strains. These five B cell epitopes cover both S1 and S2 regions. These findings are important because antibodies elicited against the S1 and S2 domains have been shown to neutralize viral infection.

In this work, we identified the putative B cell epitopes S01–S07. Of these seven epitopes, S01 (found in S1) and S05 (found in S2) are hidden within the trimer of the PEDV S glycoprotein, while the remaining epitopes (S02–S04, S06, and S07) are exposed on the surface of the trimer (according to our crystallography analysis and molecular modeling). One B cell epitope found in this study is contained within a larger neutralizing peptide described in other studies. The epitope S02 (found in S1), spanning amino acids 708–724 of the PEDV S glycoprotein, is contained within the peptide S1D, which spans amino acids 636–789. In addition, the S02 epitope is contained within one epitope reported in IEDB (ID: 2132134), while the epitope S03 had three epitopes in IEDB (ID: 2131387, ID: 2131388, and ID: 2132909). The epitopes S04, S06, and S07 were found in the S2 region. No matches were found in IEDB for epitopes S04 and S06. Surprisingly, we discovered that the S07 epitope contains the short epitope YIKWP (ID: 1330371) of the SARS-CoV-2 S glycoprotein.

We generated a construct encoding a recombinant protein that contained the exposed epitopes, in addition to the transmembrane domain from the S glycoprotein, followed by the epitopes PADRE, c-Myc, and 6Xhis at the C-terminus (Figure 3A). PK-15 cell cultures expressing the recombinant protein were selectively recognized by sera from infected pigs (Figure 3). It will be necessary to evaluate whether this display system can promote the production of neutralizing antibodies in vivo.

In addition to antibody responses, T cell responses are also important to neutralize viral infections [46,47]. T helper cell responses are desirable because they can promote antibody-based immune responses (T helper cells type 2, Th2) or cytotoxic T lymphocyte (Th1) responses that lysate virus-infected cells [46,47]. For adequate antibody production against PEDV, it is important that vaccines convey a balanced immune response based both on antibodies and T cell responses [15]. T cell epitopes are peptides composed of 7 - 17 amino acids which are produced after further processing and presented on the surface of antigen-presenting cells to activate T cells through their interaction with the T cell receptor (TCR) [21]. Thus, T cell epitopes form a complex with SLA, which is exposed on the surfaces of antigen presenting cells to trigger T cell activation. Therefore, the search for HTLs and CTLs through bioinformatics was pivotal in this work. We identified 53 likely HTL epitopes, of which 8 met all the prerequisites to be used in a molecular docking analysis, and 10 of them appeared to be promiscuous, binding to two MHC-II proteins (DRB1:0401, DRB1:1101) (Table S12). The main limitation of this section from the present study is that the NetMHCIIpan server is not yet available for swine. This is why we evaluated whether these HTL epitopes could be coupled to porcine SLA-II using molecular docking analysis. From the 53 HTL epitopes, only 8 met the requirements for further analysis with molecular docking. These 8 epitopes were coupled to SLA-II in its peptide-binding groove and occupied their main pockets (P4, P6, P7, and P9) (Figure 4) [33].

Additionally, we identified 25 CTLs in the PEDV S glycoprotein, from which 9 met all the requirements to be used in the molecular docking analysis. These 9 CTL epitopes coupled to SLA-I, and 2 CTL epitopes bound SLA-1:0401, SLA-2:0401, and SLA-3:0401 (Figure 5, Table 4 and Table 5).

Our docking analysis indicates that several predicted HTL and CTL epitopes can be coupled to SLA-II and SLA-I, respectively. Together these results highlight the ability of these epitopes to bind SLA which is one of the most important pre-requisites to activate HTLs and CTLs. It is important to emphasize that the binding of the peptide to the MHC alone is not sufficient to activate T-cell responses. Therefore, further experimental evidence, such as antigen processing, antigen presentation, cytokine production, lymphoproliferation assays, and cytotoxicity tests, will be necessary to validate any TH and CTL responses. Apparently, the humoral immune response is the most important to combat PEDV infection. Future studies should aim to elucidate the roles of both CTL and humoral immune responses to better understand their contributions in the protection against PEDV. The next step will be to construct a multiepitope-based vaccine containing both B and T cell epitopes conserved in the PEDV S glycoprotein and evaluate their antigenicity and its immunogenicity.

The development of new vaccines against PEDV could consider the use of B cell epitopes displayed on the surface of nanoparticles to facilitate the cross-linking of the B cell receptors (BCRs), which is required in the activation of B cells [48]. The administration of these five epitopes on a multimeric platform would help stimulate an adequate immune response. We have demonstrated that the display of antigens on protein particles improves the immune humoral response against viruses [49,50]. The B cell, HTL, and CTL epitopes identified here are fully conserved in Mexican PEDV strains and highly conserved in American, Asian, and European strains (Figure S5).

## 5. Conclusions

To our knowledge, this is the first report identifying B cell epitopes exposed on the surfaces of the S1 and S2 subunits from the PEDV S glycoprotein conserved in Mexican PEDV strains. In this work we developed a system to express B cell epitopes on the surfaces of transfected cells using a signal peptide and a transmembrane domain derived from the PEDV S glycoprotein. The fact that the predicted HTL and CTL epitopes meet all selection criteria and fit into pockets located in the peptide-binding groove of SLA-I and SLA-II is very encouraging. These conserved epitopes are expected to elicit antibody, HTL, and CTL responses; the latter is rarely or not considered in PEDV vaccine development. Further experimental analysis of the immune response generated by these B and T cell epitopes is warranted. These epitope candidates should be evaluated in depth including an analysis of cytokines production, cytotoxic and neutralizing effects

## Figures and Tables

**Table 1:** Conserved B cell epitopes of the S glycoprotein of the Mexican PEDV strains, their localization, and their amino acid sequences.

Epitope	Structural Localization	Location of Amino Acids	Amino Acid Sequence
S01	Hidden	279–286	VSNQPLLV
S02	Exposed	708–724	PCSFSEQAAYVDDDIVG
S03	Exposed	792–832	SIPTNFSMSIRTEYLQLYNTPVSVDCATYVCNGNSRCKQLL
S04	Exposed	984–988	SYAVQA
S05	Hidden	1064–1073	QLQHNFQAIS
S06	Exposed	1181–1189	LCVNDEIAL
S07	Exposed	1310–1336	QSLIYNINNTLVDLEWLNRVETYIKWP

**Table 2:** HTL epitopes derived from PEDV S glycoprotein that met criteria in the antigenicity, allergenicity, autoimmunity, and toxicity analyses.

Epitope	Amino Acid Sequence	VaxiJen	AllerTOP	Autoimmunity	ToxinPred
HTL01	VTYVNLTRDQLP	0.6783	No	No	Non toxin
HTL09	RTEYLQLYNTPVS	0.5236	No	No	Non toxin
HTL12	EGSIVLHTALGTN	0.5270	No	No	Non toxin
HTL13	PGVVDAEKLHMYS	0.7461	No	No	Non toxin
HTL17	RLQPYEVFEKVHVQ	0.6110	No	No	Non toxin
HTL27	QLTVQLQHNFQAIS	1.2204	No *	No	Non toxin
HTL41	QAAYVDDDIVGVISS	0.5450	No	No	Non toxin
HTL49	LAEGSIVLHTALGTN	0.5385	No	No	Non toxin

HTL epitopes with a threshold ≥0.5 are ranked according to their antigenic value using the VaxiJen server. * No significant similarity.

**Table 3:** Free energy of coupling between HTL epitopes and SLA-II molecules.

Epitope	Amino Acid Sequence	ΔG (Kcal/mol) with SLA-DRB1:0401	ΔG (Kcal/mol) with SLA-DRB1:1101	ΔG (Kcal/mol) with SLA-DRB1:1301
HTL01	VTYVNLTRDQLP	−8.4	−8.4	−8.6
HTL09	RTEYLQLYNTPVS	−9.4	−9.2	−7.5
HTL12	EGSIVLHTALGTN	−9.1	−8.8	−8.4
HTL13	PGVVDAEKLHMYS	−7.1	−7.5	−7.7
HTL17	RLQPYEVFEKVHVQ	−8.1	−8.7	−8.5
HTL27	QLTVQLQHNFQAIS	−10.7	−9.1	−8.6
HTL41	QAAYVDDDIVGVISS	−8.8	−9.1	−8.9
HTL49	LAEGSIVLHTALGTN	−8.6	−8.9	−8.4

**Table 4:** Prediction of CTL epitopes derived from PEDV S glycoprotein that met criteria in the allergenicity, antigenicity, toxicity, and autoimmunity analyses.

Epitope	Core	VaxiJen	AllerTOP	Autoimmunity	ToxinPred
CTL06	AIGNITSAF	0.5398	No	No	Non toxin
CTL22	ISIPTNFSM	0.6356	No	No	Non toxin
CTL20	NRVETYIKW	0.7349	No	No	Non toxin
CTL05	VVDAEKLHM	0.7773	Probable allergen *	No	Non toxin
CTL12	CSFSEQAAY	0.8145	No	No	Non toxin
CTL17	YAVQARLNY	1.0482	Probable allergen *	No	Non toxin
CTL04	VADLVCAQY	1.228	No	No	Non toxin
CTL18	LTVQLQHNF	1.2798	No	No	Non toxin
CTL03	FSMSIRTEY	1.5744	Probable allergen *	No	Non toxin

Prediction of CTL epitopes with a threshold ≥0.5, which are ranked according to their antigenic value using the VaxiJen server. Promiscuous epitopes are highlighted in magenta. * No significant similarity.

**Table 5:** Free energy of coupling of predicted CTL epitopes and SLA-I molecules.

Epitope	Conserved Amino Acid Sequence	Allele (NetMHCPan)	Peptide	ΔG (Kcal/mol) with SLA-1:0401	ΔG (Kcal/mol) with SLA-2:0401	ΔG (Kcal/mol) with SLA-3:0401
CTL17	AAALPFSYAVQARLNY	SLA-2:0401	YAVQARLNY	−12.1	−10.7	−10.6
CTL03	TGNISIPTNFSMSIRTEYLQLYNTPVSVDCATYVCNGNSRCKQLLTQY	SLA-2:0401	FSMSIRTEY	−11.2	−8.2	−10.3
CTL18	QLTVQLQHNFQAISSSI	SLA-2:0401	LTVQLQHNF	−11.1	−8.7	−8.8
CTL04	KRSFIEDLLFNKVVTNGLGTVDEDYKRCSNGRSVADLVCAQYYSGVMVLPGVVDAEKLHMYSASLIGGMVLGG	SLA-1:0401	VADLVCAQY	−10.9	−8.6	−10.1
CTL06	ALQTDVLQRNQQLLAESFNSAIGNITSAFESVKEA	SLA-1:0401	AIGNITSAF	−10.9	−9.8	−10.5
CTL20	TEELQSLIYNINNTLVDLEWLNRVETYIKWPWWVWLIIFIVLIFVVSLLVFCCISTGCCGCCGCCCACF	SLA-3:0401	NRVETYIKW	−10.9	−8.6	−8.1
CTL05	KRSFIEDLLFNKVVTNGLGTVDEDYKRCSNGRSVADLVCAQYYSGVMVLPGVVDAEKLHMYSASLIGGMVLGG	SLA-1:0401	VVDAEKLHM	−10.3	−8.9	−9.7
CTL22	TGNISIPTNFSMSIRTEYLQLYNTPVSVDCATYVCNGNSRCKQLLTQY	SLA-3:0401	ISIPTNFSM	−10.2	−9.3	−9.3
CTL12	VYSVTPCSFSEQAAYVDDDIVGVISS	SLA-2:0401	CSFSEQAAY	−10.2	−8.9	−9.9

Predicted CTL epitopes that bind to SLA-1*401, SLA-2*401, and SLA-3*401 with the lowest free energy (ΔG). All the nine peptides bind to SLA-I with low free energy. Promiscuous epitopes (FSMSIRTEY and NRVETYIKW) are highlighted in magenta.

## Data Availability

The data used in this research can be found in the GenBank database. For more information, please contact the corresponding author.

## Supplementary Materials

The Supplementary Tables and Figures can be downloaded at: https://www.mdpi.com/article/10.3390/v18040407/s1.

## Abbreviations

The following abbreviations are used in this manuscript:

2C10	2C10 peptide of PEDV
BLAST	Basic Local Alignment Search Tool
COE	CO-26K-Equivalent peptide of PEDV
CTL	Cytotoxic T lymphocyte
E	E protein
G1	PEDV genotype 1
G1a	PEDV subgenotype 1a
G2	PEDV genotype 2
G2b	PEDV subgenotype 2b
GI	Classical PEDV strains
GII	Mutant PEDV strains
GRAVY	Grand Average Hydropathicity
HTL	Helper T lymphocyte
IgG	Immunoglobulin G
IgM	Immunoglobulin M
INDEL	Insertion-Deletion
IPD	Immuno Polymorphism Database
kb	kilobase
kDa	kilodalton
M	M protein
MAFFT	Multiple Alignment Using Fast Fourier Transform
MEGA11	Molecular Evolutionary Genetics Analysis version 11
MHC	Major Histocompatibility Complex
mRNA	Messenger Ribonucleic Acid
myc	Peptide EQKLISEEDL
N	N protein
nm	nanometer
ORF	Open Reading Frame
PADRE	Pan-DR Epitope
PDB	Protein Data Bank
PEDV	Porcine Epidemic Diarrhea Virus
R	Recombinant genotypes of PEDV
RBD	Receptor Binding Domain
S	Spike protein
S1	Subunit 1 from Glycoprotein S
S1D	Residues 636–789 of the Spike Glycoprotein
S2	Subunit 2 from Glycoprotein S
SIgA	Secretory Immunoglobulin A
SLA	Swine Leukocyte Antigens
SP	Signal Peptide
SS2	Peptide YSNIGVCK
SS6	Peptide LQDGQVKI
TM	Transmembrane
US	United States
