# Supplementary material for: Identification of Conserved B and T Cell Epitopes in Glycoprotein S of Mexican Porcine Epidemic Diarrhea Virus (PEDV) Strains via Immunoinformatics Analysis, Molecular Docking, and Immunofluorescence
Source: Viruses. 2026 Mar 25;18(4):407. doi: 10.3390/v18040407 (PMC13120105; doi:10.3390/v18040407)
Supplement: Supplementary file 1 [file viruses-18-00407-s001.zip › Figure S1 PROCHECK analysisof glycoprotein models.pdf]

a) Mich(2013)MH006965.1

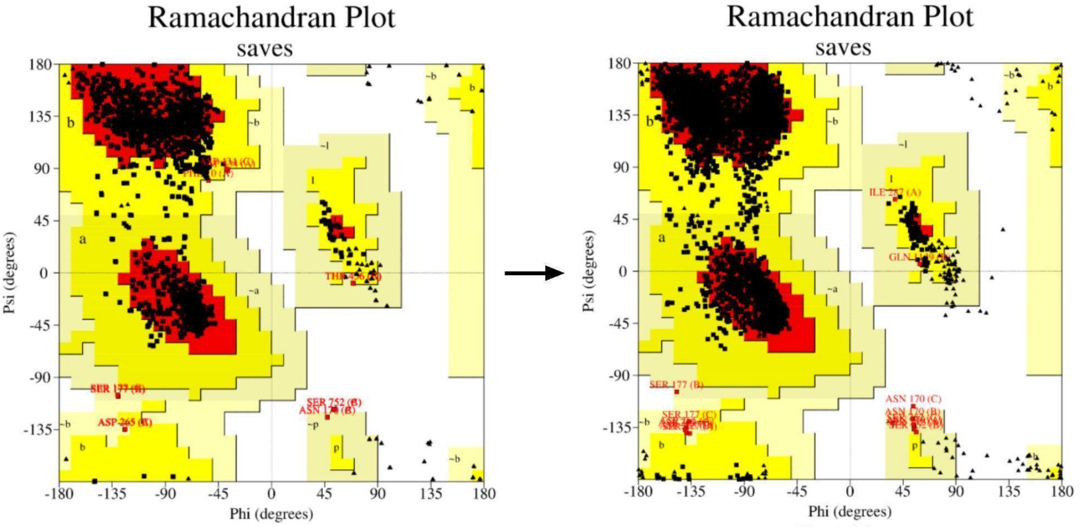

Plot statistics

Residues in most favoured regions [A,B,L]	3348	90.5%
Residues in additional allowed regions [a,b,l,p]	333	9.0%
Residues in generously allowed regions [~a,~b,~l,~p]	15	0.4%
Residues in disallowed regions	3	0.1%
	----	------
Number of non-glycine and non-proline residues	3699	100.0%
Number of end-residues (excl. Gly and Pro)	6	
Number of glycine residues (shown as triangles)	291	
Number of proline residues	162	

Total number of residues	4158	

Plot statistics

Residues in most favoured regions [A,B,L]	3379	91.3%
Residues in additional allowed regions [a,b,l,p]	306	8.3%
Residues in generously allowed regions [~a,~b,~l,~p]	13	0.4%
Residues in disallowed regions	1	0.0%
	----	------
Number of non-glycine and non-proline residues	3699	100.0%
Number of end-residues (excl. Gly and Pro)	6	
Number of glycine residues (shown as triangles)	291	
Number of proline residues	162	

Total number of residues	4158	

b) Tlax(2014)MN091346.1

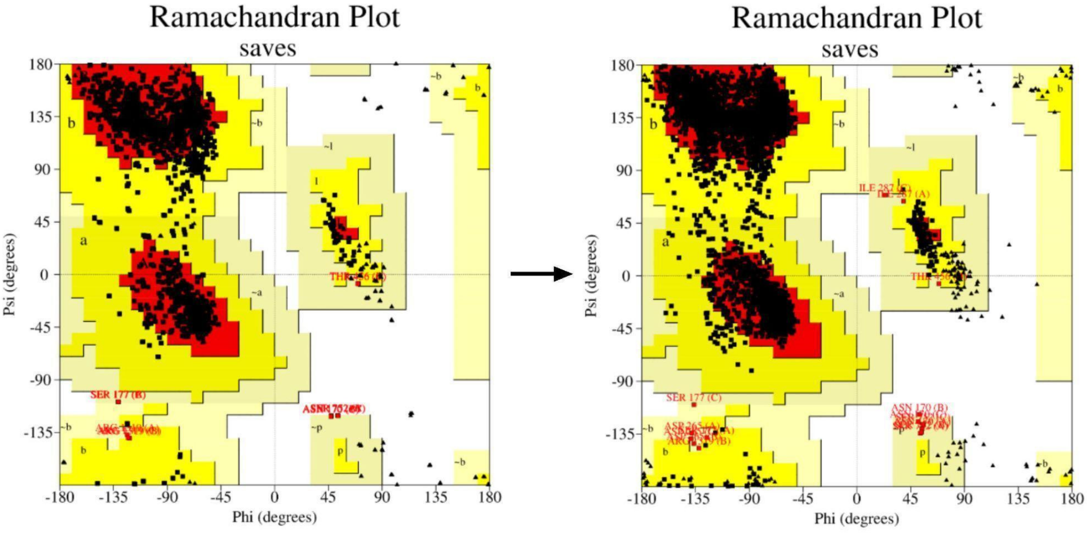

Plot statistics

Residues in most favoured regions [A,B,L]	3337	90.1%
Residues in additional allowed regions [a,b,l,p]	351	9.5%
Residues in generously allowed regions [~a,~b,~l,~p]	14	0.4%
Residues in disallowed regions	0	0.0%
	----	------
Number of non-glycine and non-proline residues	3702	100.0%
Number of end-residues (excl. Gly and Pro)	6	
Number of glycine residues (shown as triangles)	291	
Number of proline residues	159	

Total number of residues	4158	

Plot statistics

Residues in most favoured regions [A,B,L]	3378	91.2%
Residues in additional allowed regions [a,b,l,p]	309	8.3%
Residues in generously allowed regions [~a,~b,~l,~p]	14	0.4%
Residues in disallowed regions	1	0.0%
	----	------
Number of non-glycine and non-proline residues	3702	100.0%
Number of end-residues (excl. Gly and Pro)	6	
Number of glycine residues (shown as triangles)	291	
Number of proline residues	159	

Total number of residues	4158	

c) Ver(2015)MH013464.1

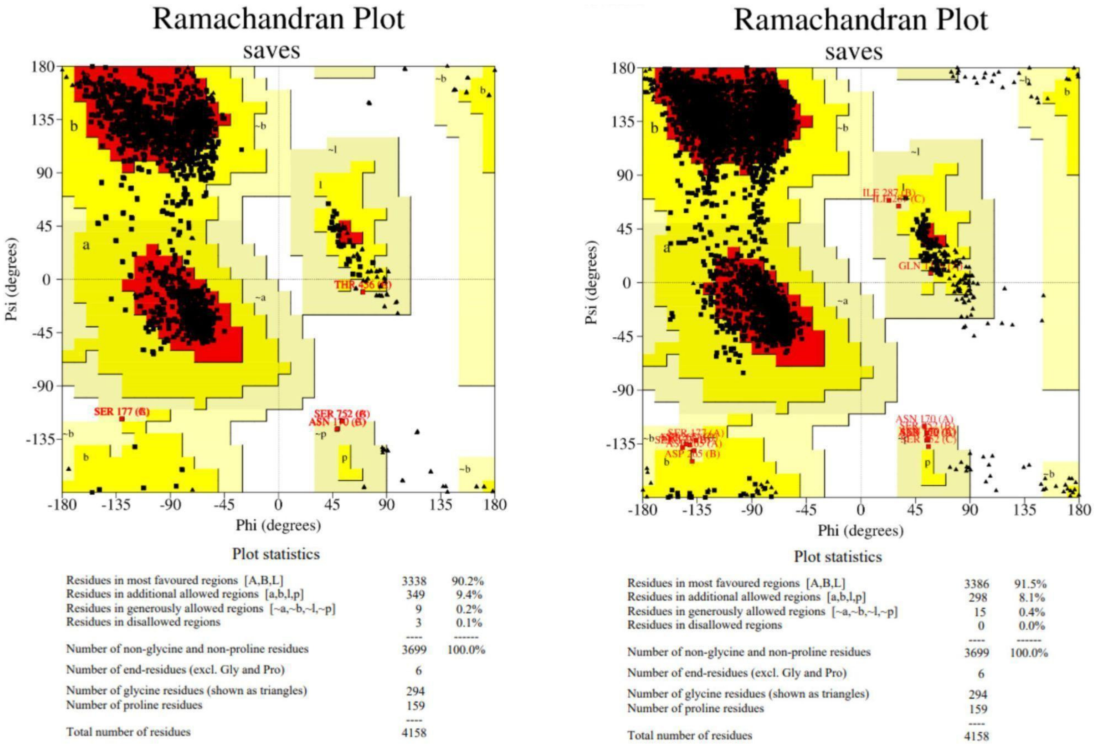

d) Pue(2016)MH006963.1

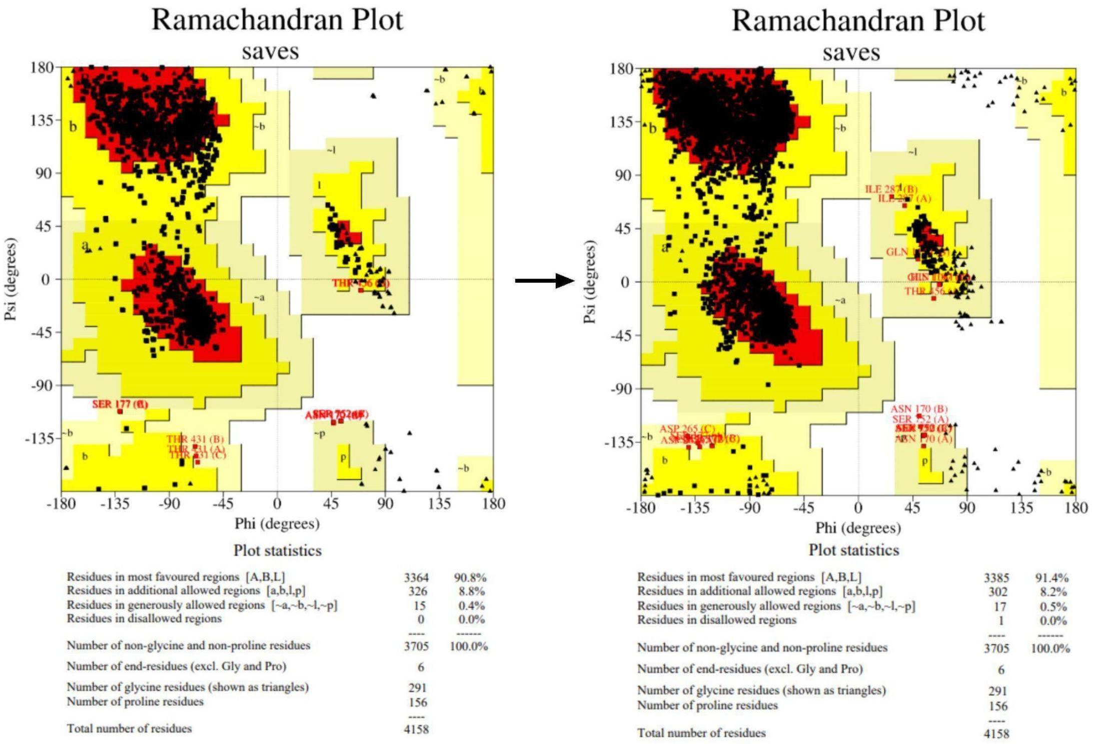

e) Jal(2017)MH004420.1

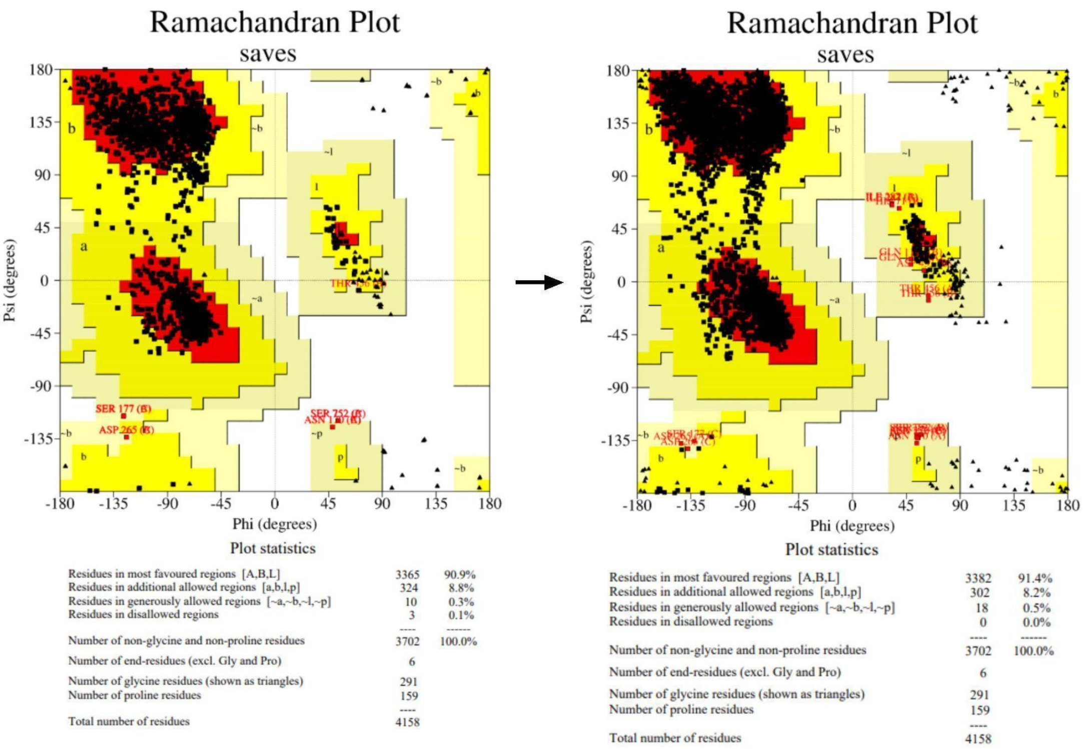

f) EdoMex(2018)MT490316.1

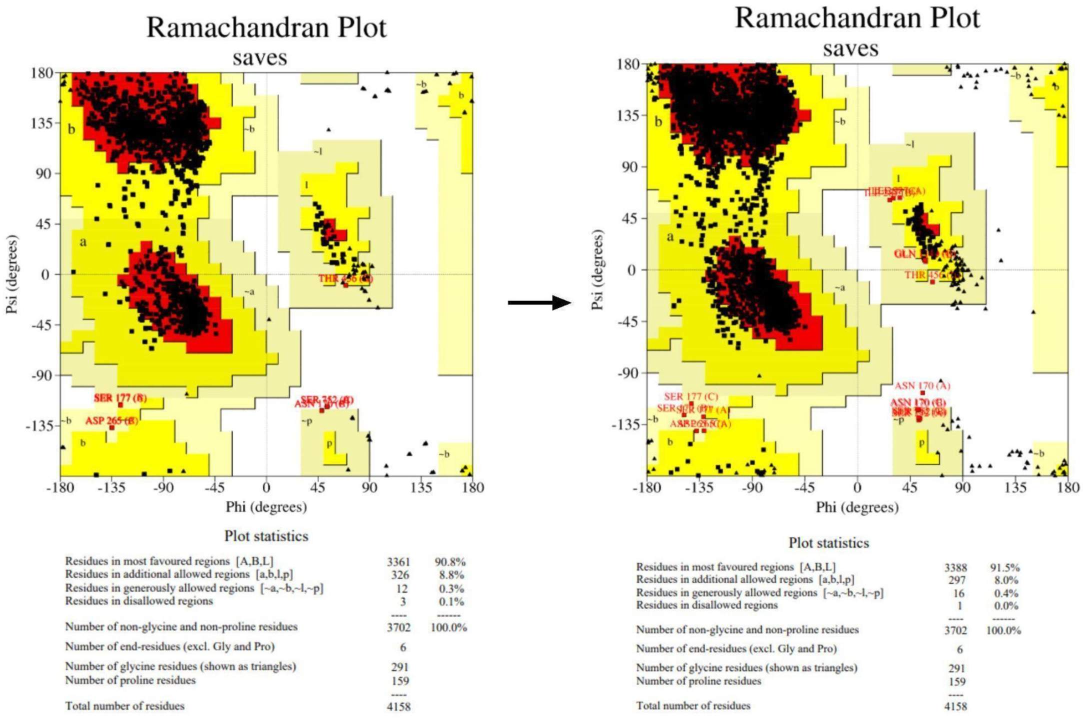

Figure S1. Validation of glycoprotein S models from Mexican PEDV strains.

The Ramachandran plots show the residues (dots) of models before refinement (left) and after refinement (right) in most favored regions (red), additional allowed regions (bright yellow), generously allowed regions (light yellow), and disallowed

regions (white). **a)** Mich(2013)MH006965.1: the model before refinement had 90.5% favored regions and after refinement it increased to 91.3%. **b)** Tlax(2014)MN091346.1: the model before refinement had 90.1% favored regions and after refinement it increased to 91.2%. **c)** Ver(2015)MH013464.1: the model before refinement had 90.2% favored regions and after refinement it increased to 91.5%. **d)** Pue(2016)MH006963.1: the model before refinement had 90.8% favored regions and after refinement it increased to 91.4%. **e)** Jal(2017)MH004420.1: the model before refinement had 90.9% favored regions and after refinement it increased to 91.4%. **f)** EdoMex(2018)MT490316.1: the model before refinement had 90.8% favored regions and after refinement it increased to 91.5%.
