# Supplementary material for: Identification of Conserved B and T Cell Epitopes in Glycoprotein S of Mexican Porcine Epidemic Diarrhea Virus (PEDV) Strains via Immunoinformatics Analysis, Molecular Docking, and Immunofluorescence
Source: Viruses. 2026 Mar 25;18(4):407. doi: 10.3390/v18040407 (PMC13120105; doi:10.3390/v18040407)
Supplement: Supplementary file 1 [file viruses-18-00407-s001.zip › Figure S2 Validation of predicted SLA-II models.pdf]

A) **SLA-DRB1*0401**

Before refining

After refining

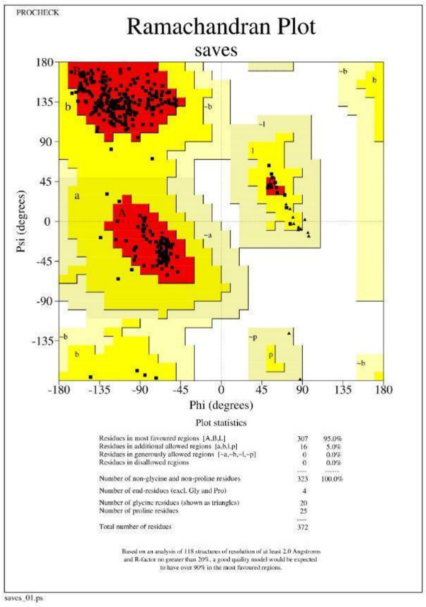

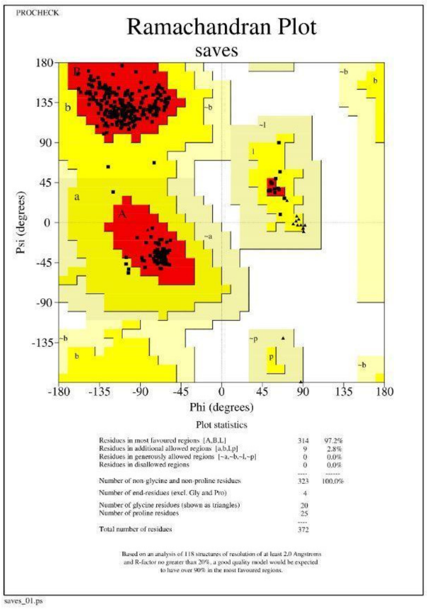

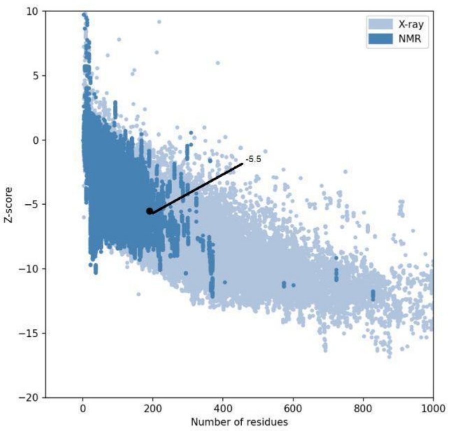

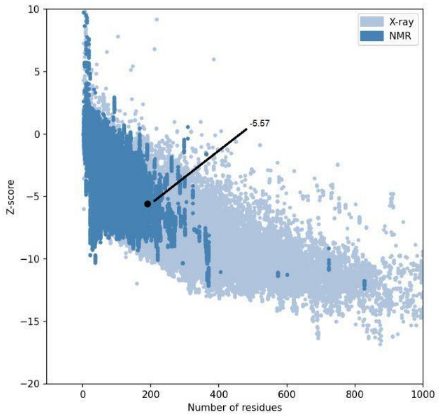

B) **SLA-DRB1*1101**

Before refining

After refining

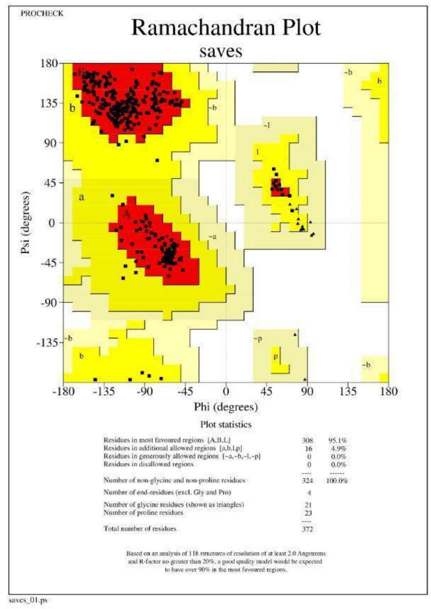

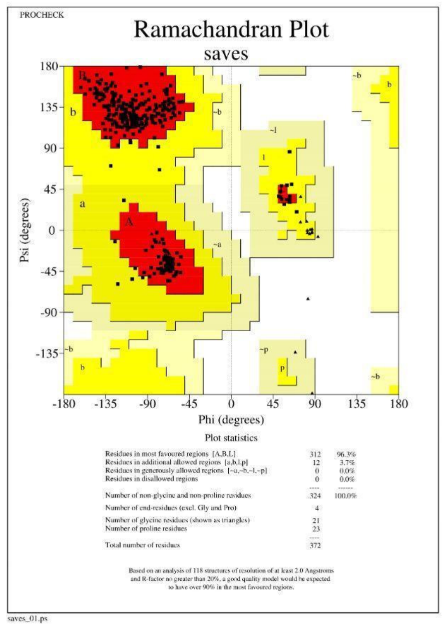

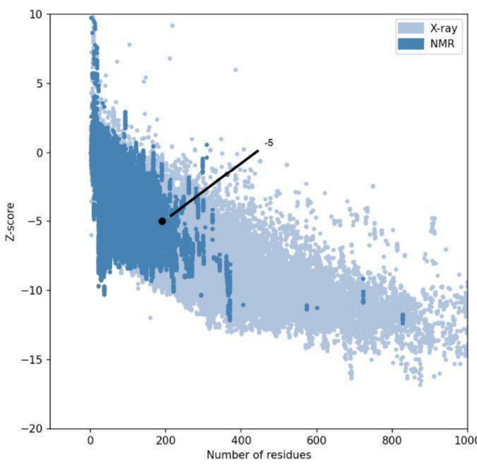

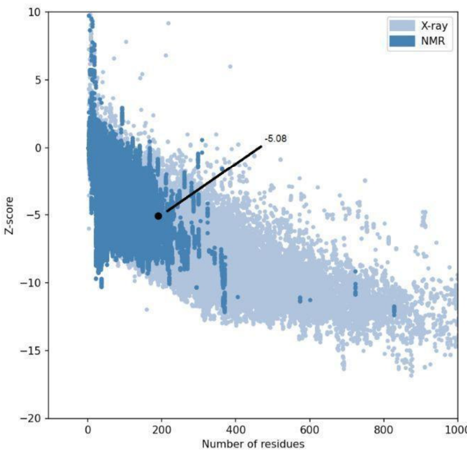

C) **SLA-DRB1*1301**

Before refining **After refining**

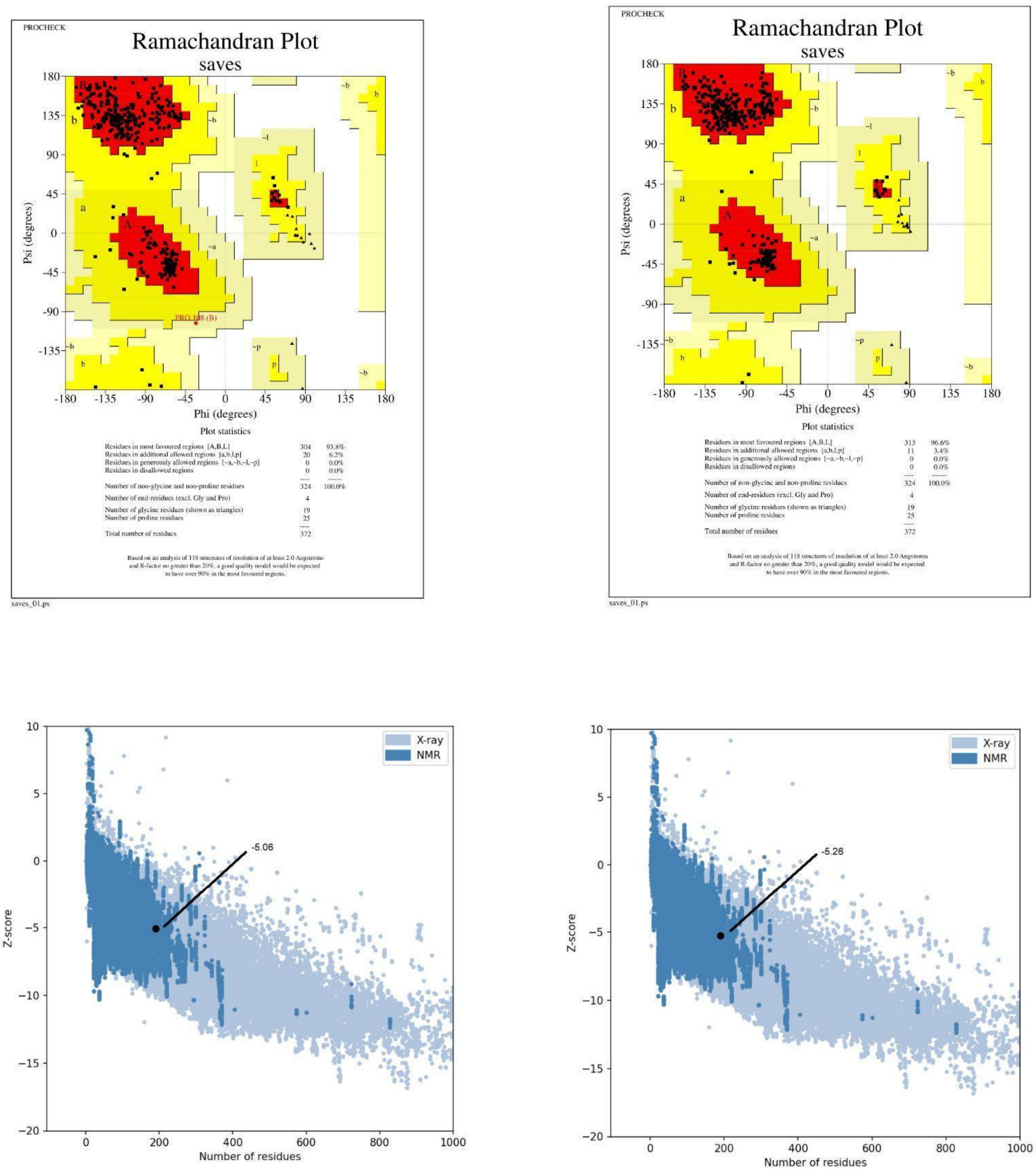

Figure S2. Validation of predicted SLA-II models. The plots show the residues (dots) of models before refinement (left) and after refinement (right) in most favored regions (red), additional allowed regions (bright yellow), generously allowed regions (light yellow), disallowed regions (white), and the Z-score used to validate the predicted MHC models. A) SLA-DRB1*0401. The model before refinement had 95% favored regions and after refinement it increased to 97.2%. The Z-score decreased from -5.5 to -5.57 (ipTM: 0.92; pTM: 0.91); B) SLA-DRB1*1101. The model before refinement had 95.1% favored regions and after refinement it increased to 96.3%. The Z-score decreased from -5 to -5.08 (ipTM: 0.88; pTM: 0.88); C) SLA-DRB1*1301 The

model before refinement had 93.8% favored regions, and after refinement this increased to 96.6%. The Z-score decreased from -5.06 to -5.26 (ipTM: 0.89; pTM: 0.89). *ipTM: interface-pTM; *pTM: Predicted Template Modeling. *Obtained from structural analysis with AlphaFold server (V. 3).
