# Supplementary material for: Identification of Conserved B and T Cell Epitopes in Glycoprotein S of Mexican Porcine Epidemic Diarrhea Virus (PEDV) Strains via Immunoinformatics Analysis, Molecular Docking, and Immunofluorescence
Source: Viruses. 2026 Mar 25;18(4):407. doi: 10.3390/v18040407 (PMC13120105; doi:10.3390/v18040407)
Supplement: Supplementary file 1 [file viruses-18-00407-s001.zip › Figure S3 Validation of predicted SLA-I models.pdf]

A) SLA-2*0401

Before refining

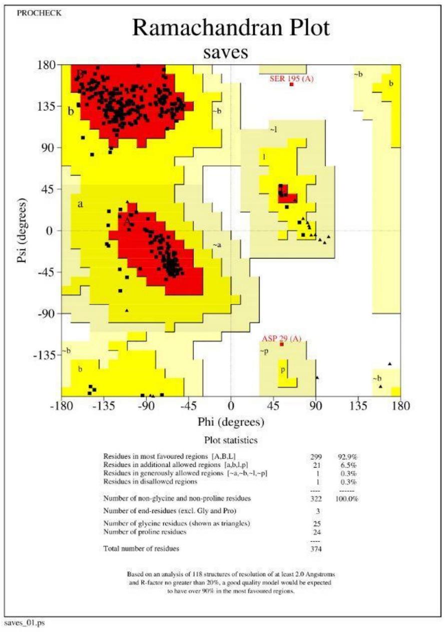

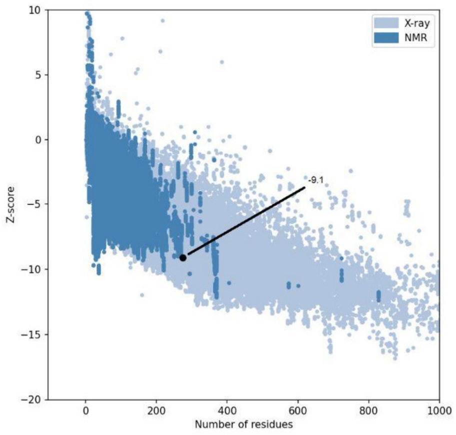

After refining

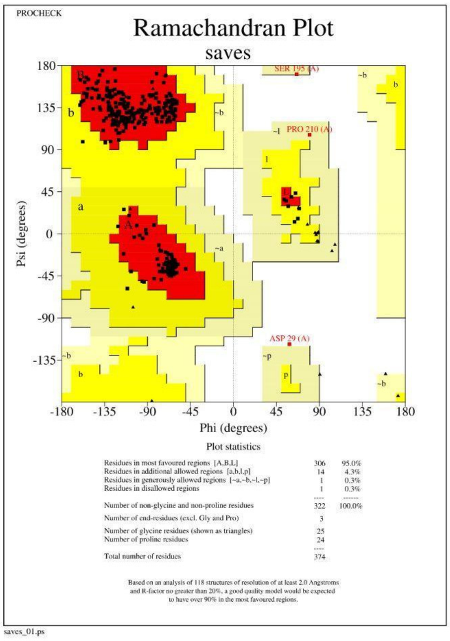

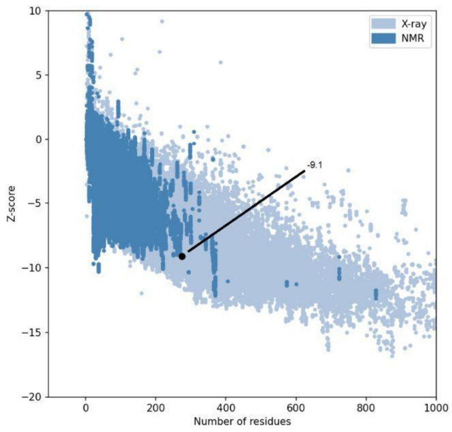

B) SLA-3*0401

Before refining **After refining**

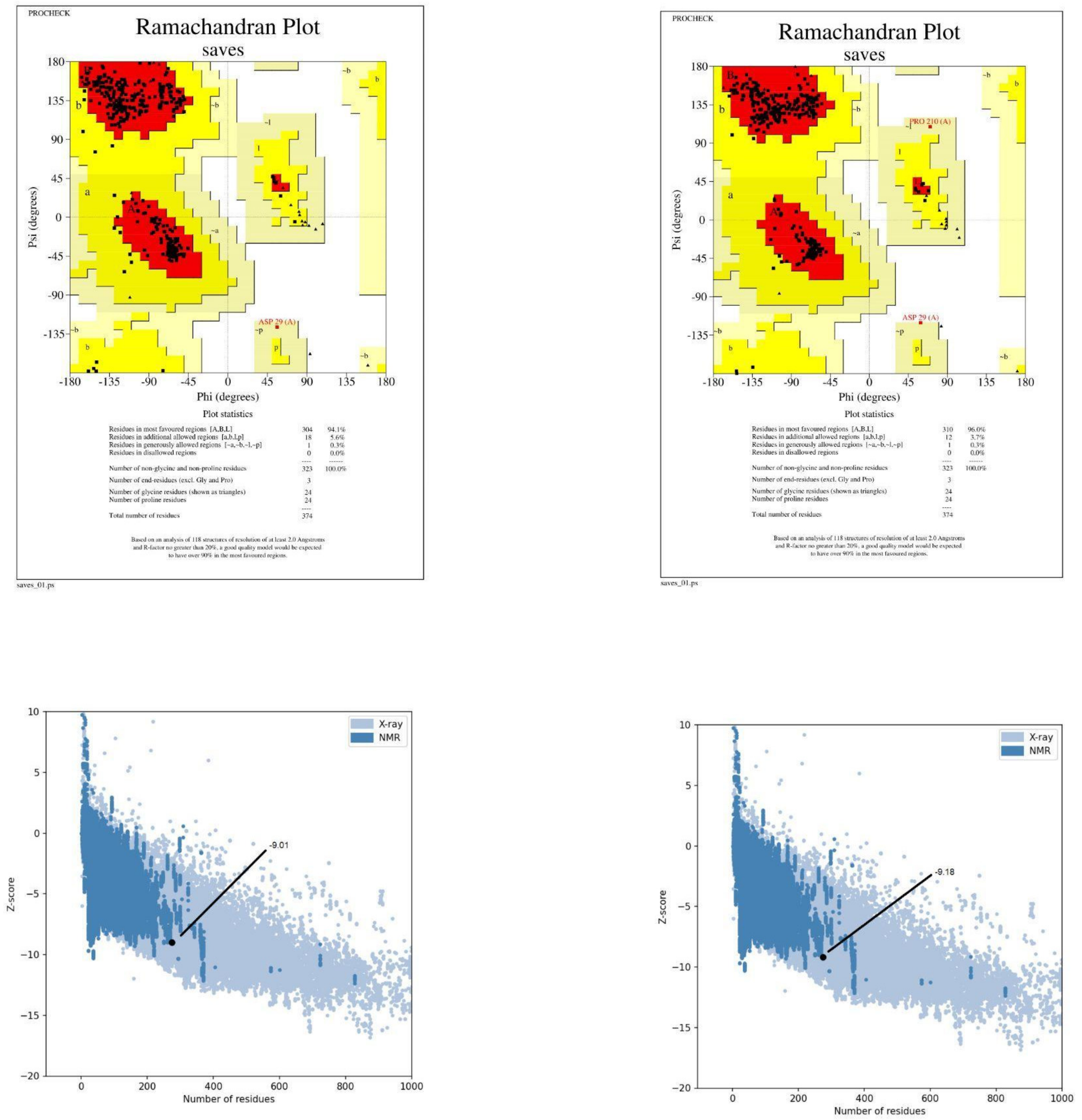

Figure S3. Validation of predicted MHC models. The plots show the residues (dots) of models before refinement (left) and after refinement (right) in most favored regions (red), additional allowed regions (bright yellow), generously allowed regions (light yellow), disallowed regions (white), and the Z-score for validates the predicted MHC models. A) SLA-2*0401 (ipTM: 0.94; pTM: 0.93). The model before refinement had 92.9% favored regions and after refinement it increased to 95%. The Z-score remained at -9.1; B) SLA-3*0401. The model before refinement had 94.1% favored regions and after refinement it increased to 96%. The Z-score decreased from -9.01 to -9.18 (ipTM: 0.94; pTM: 0.93). *ipTM: interface-pTM; *pTM: Predicted Template Modeling. *Obtained from structural analysis with AlphaFold server (V. 3).
