# Supplementary material for: Identification of Conserved B and T Cell Epitopes in Glycoprotein S of Mexican Porcine Epidemic Diarrhea Virus (PEDV) Strains via Immunoinformatics Analysis, Molecular Docking, and Immunofluorescence
Source: Viruses. 2026 Mar 25;18(4):407. doi: 10.3390/v18040407 (PMC13120105; doi:10.3390/v18040407)
Supplement: Supplementary file 1 [file viruses-18-00407-s001.zip › Figure S4 Alignment with reported peptides from S glycoprotein.pdf]

A) Alignment of the COE region

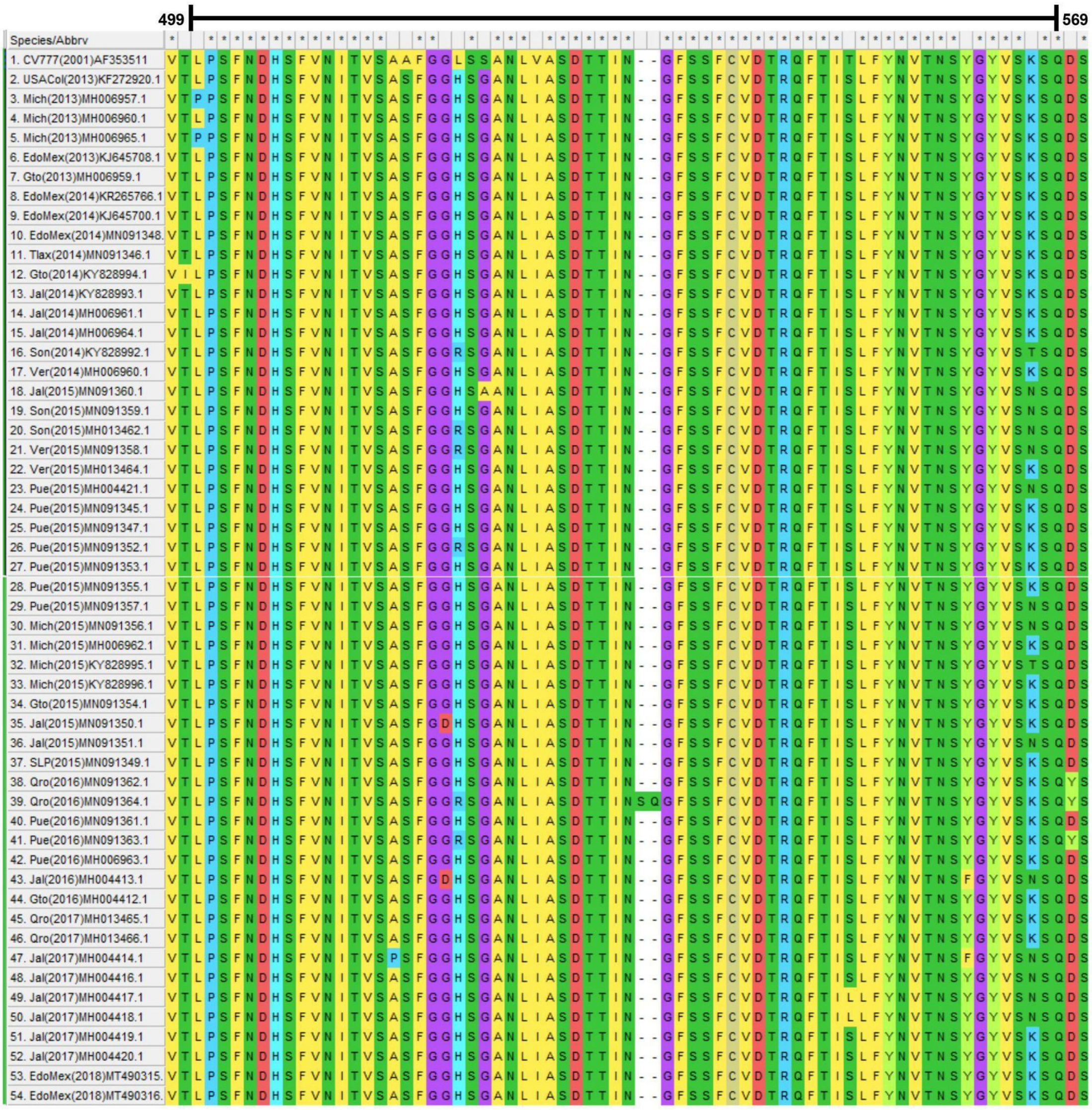

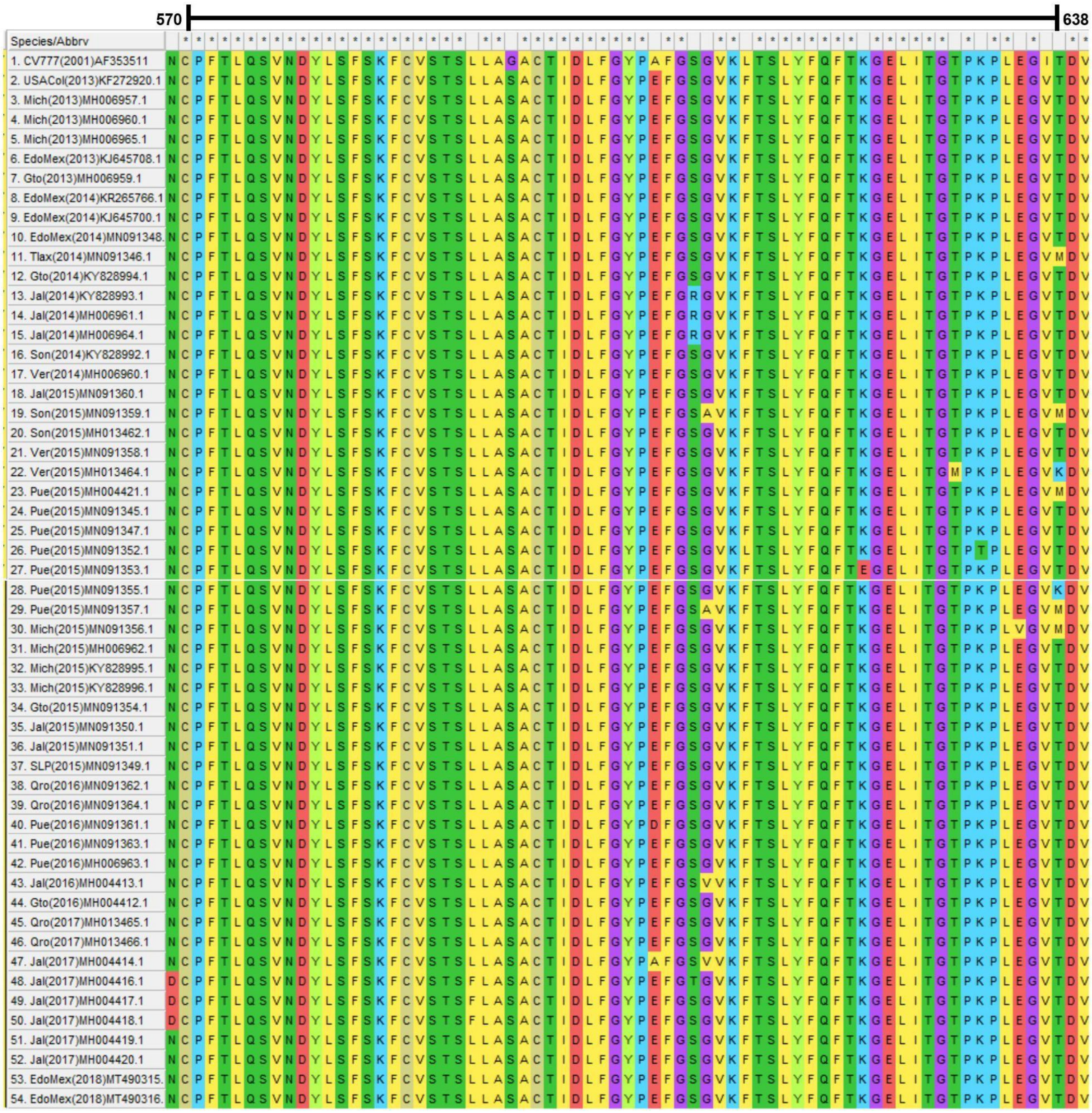
570
638
Species/Abbrv
1. CV777(2001)AF353511
2. USACol(2013)KF272920.1
3. Mich(2013)MH006957.1
4. Mich(2013)MH006960.1
5. Mich(2013)MH006965.1
6. EdoMex(2013)KJ645708.1
7. Gto(2013)MH006959.1
8. EdoMex(2014)KR265766.1
9. EdoMex(2014)KJ645700.1
10. EdoMex(2014)MN091348.
11. Tlax(2014)MN091346.1
12. Gto(2014)KY828994.1
13. Jal(2014)KY828993.1
14. Jal(2014)MH006961.1
15. Jal(2014)MH006964.1
16. Son(2014)KY828992.1
17. Ver(2014)MH006960.1
18. Jal(2015)MN091360.1
19. Son(2015)MN091359.1
20. Son(2015)MH013462.1
21. Ver(2015)MN091358.1
22. Ver(2015)MH013464.1
23. Pue(2015)MH004421.1
24. Pue(2015)MN091345.1
25. Pue(2015)MN091347.1
26. Pue(2015)MN091352.1
27. Pue(2015)MN091353.1
28. Pue(2015)MN091355.1
29. Pue(2015)MN091357.1
30. Mich(2015)MN091356.1
31. Mich(2015)MH006962.1
32. Mich(2015)KY828995.1
33. Mich(2015)KY828996.1
34. Gto(2015)MN091354.1
35. Jal(2015)MN091350.1
36. Jal(2015)MN091351.1
37. SLP(2015)MN091349.1
38. Qro(2016)MN091362.1
39. Qro(2016)MN091364.1
40. Pue(2016)MN091361.1
41. Pue(2016)MN091363.1
42. Pue(2016)MH006963.1
43. Jal(2016)MH004413.1
44. Gto(2016)MH004412.1
45. Qro(2017)MH013465.1
46. Qro(2017)MH013466.1
47. Jal(2017)MH004414.1
48. Jal(2017)MH004416.1
49. Jal(2017)MH004417.1
50. Jal(2017)MH004418.1
51. Jal(2017)MH004419.1
52. Jal(2017)MH004420.1
53. EdoMex(2018)MT490315.
54. EdoMex(2018)MT490316.

B) Alignment of the S1D region

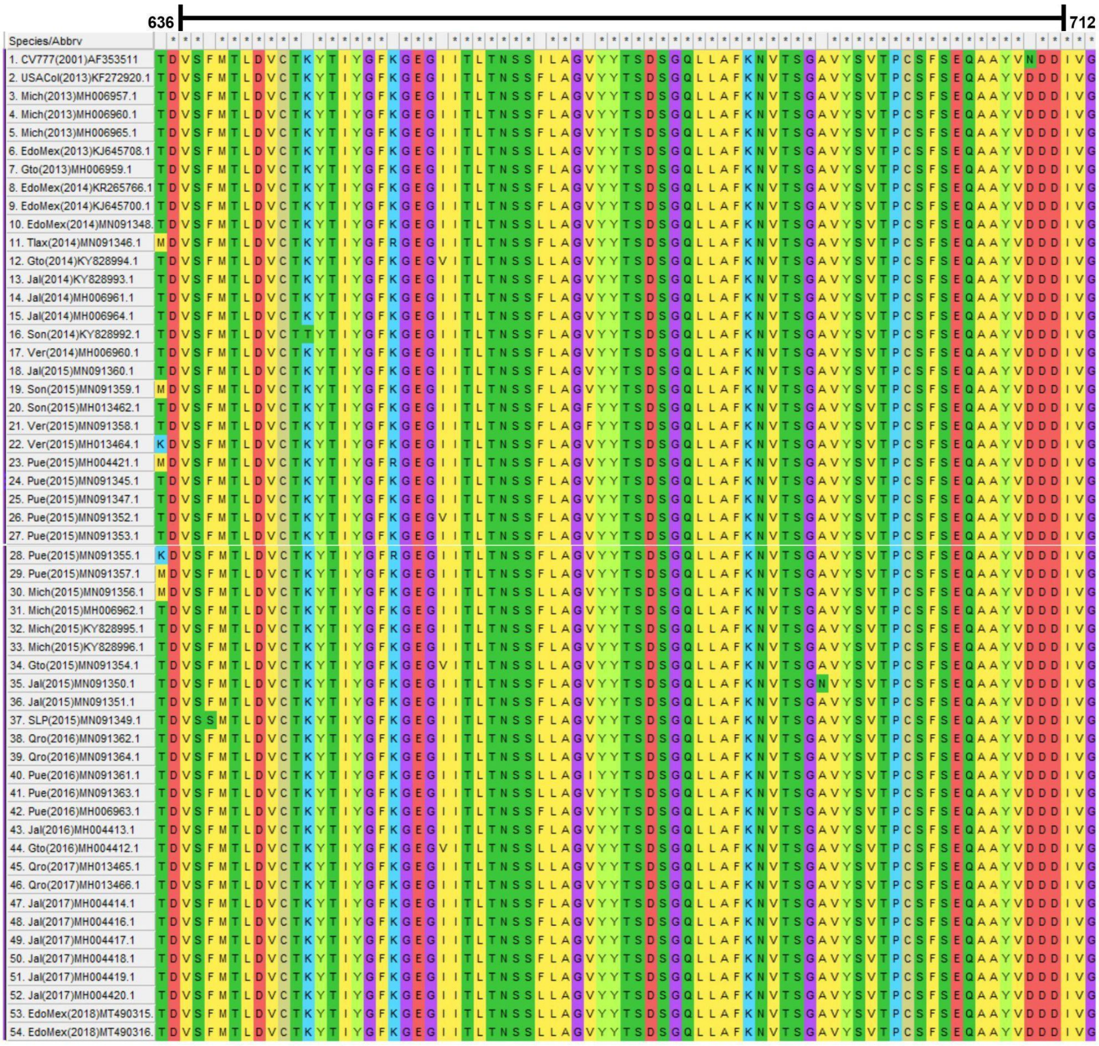

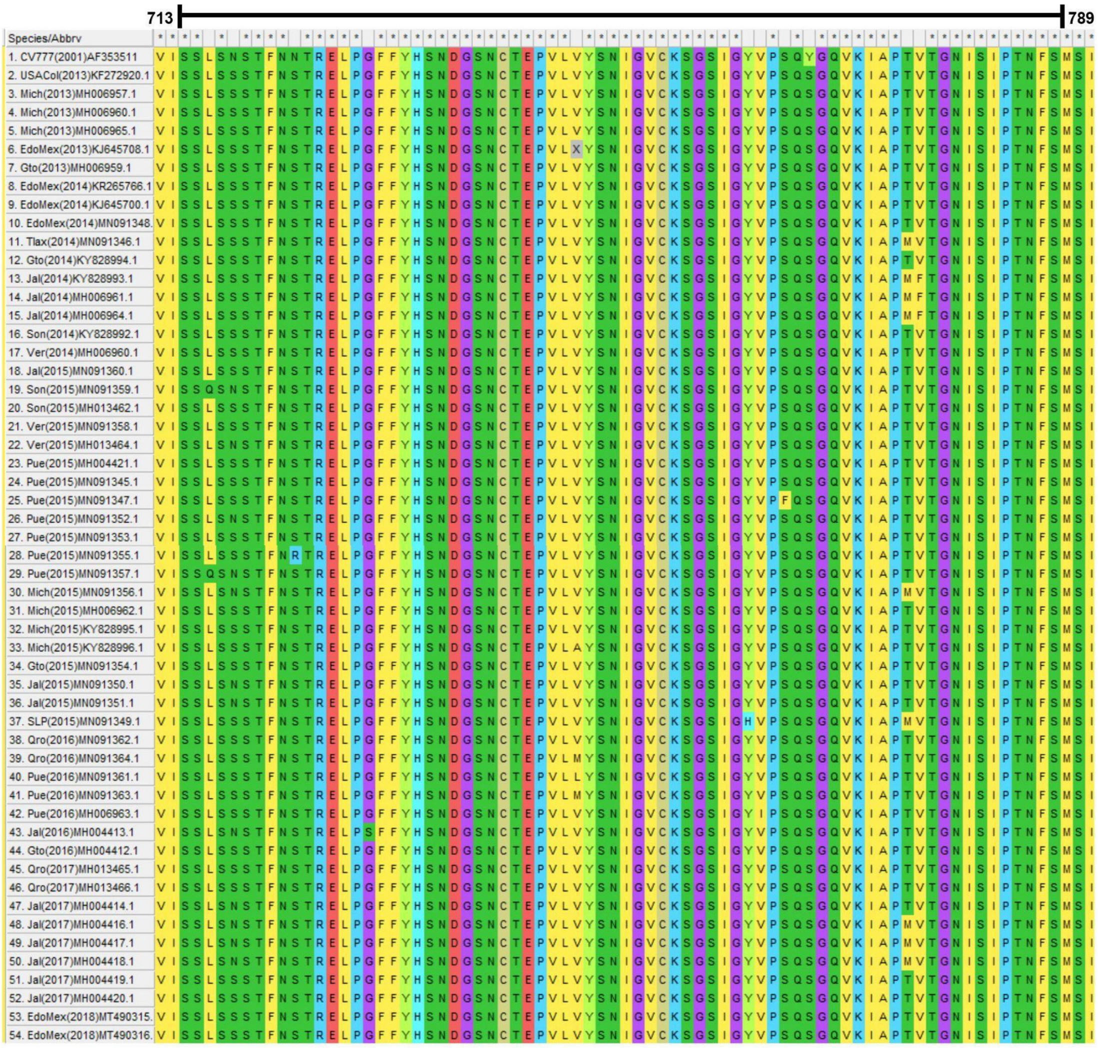
713
789
Species/Abbrv
1. CV777(2001)AF353511
2. USACol(2013)KF272920.1
3. Mich(2013)MH006957.1
4. Mich(2013)MH006960.1
5. Mich(2013)MH006965.1
6. EdoMex(2013)KJ645708.1
7. Gto(2013)MH006959.1
8. EdoMex(2014)KR265766.1
9. EdoMex(2014)KJ645700.1
10. EdoMex(2014)MN091348.
11. Tlax(2014)MN091346.1
12. Gto(2014)KY828994.1
13. Jal(2014)KY828993.1
14. Jal(2014)MH006961.1
15. Jal(2014)MH006964.1
16. Son(2014)KY828992.1
17. Ver(2014)MH006960.1
18. Jal(2015)MN091360.1
19. Son(2015)MN091359.1
20. Son(2015)MN013462.1
21. Ver(2015)MN091358.1
22. Ver(2015)MH013464.1
23. Pue(2015)MH004421.1
24. Pue(2015)MN091345.1
25. Pue(2015)MN091347.1
26. Pue(2015)MN091352.1
27. Pue(2015)MN091353.1
28. Pue(2015)MN091355.1
29. Pue(2015)MN091357.1
30. Mich(2015)MN091356.1
31. Mich(2015)MH006962.1
32. Mich(2015)KY828995.1
33. Mich(2015)KY828996.1
34. Gto(2015)MN091354.1
35. Jal(2015)MN091350.1
36. Jal(2015)MN091351.1
37. SLP(2015)MN091349.1
38. Qro(2016)MN091362.1
39. Qro(2016)MN091364.1
40. Pue(2016)MN091361.1
41. Pue(2016)MN091363.1
42. Pue(2016)MH006963.1
43. Jal(2016)MH004413.1
44. Gto(2016)MH004412.1
45. Qro(2017)MH013465.1
46. Qro(2017)MH013466.1
47. Jal(2017)MH004414.1
48. Jal(2017)MH004416.1
49. Jal(2017)MH004417.1
50. Jal(2017)MH004418.1
51. Jal(2017)MH004419.1
52. Jal(2017)MH004420.1
53. EdoMex(2018)MT490315.
54. EdoMex(2018)MT490316.

C) Alignment of the 2C10 region

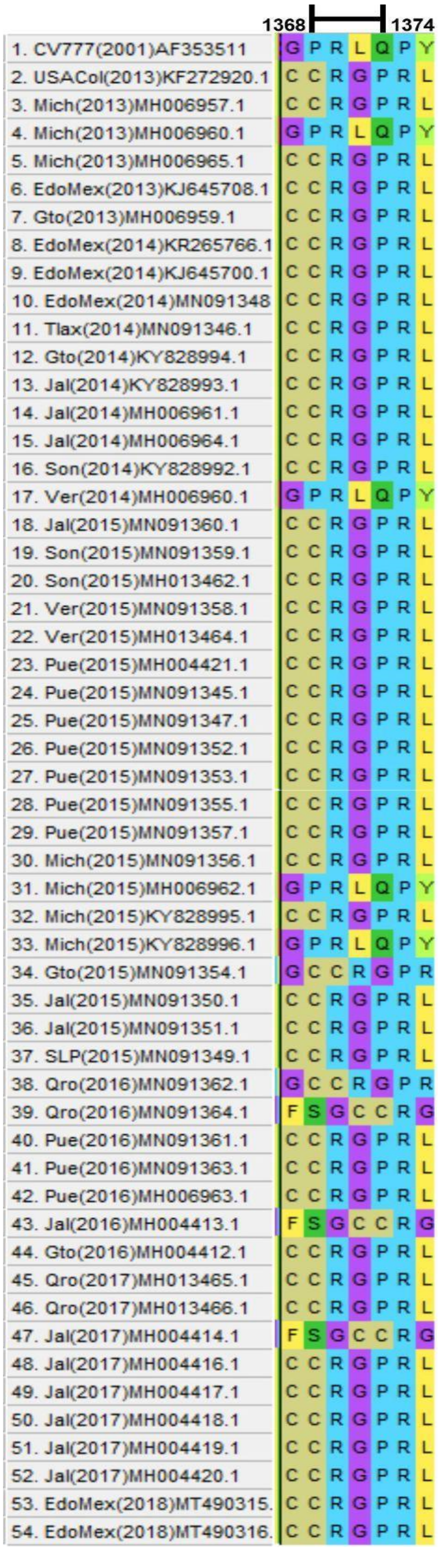

D) Alignment of the SS2 and SS4 region

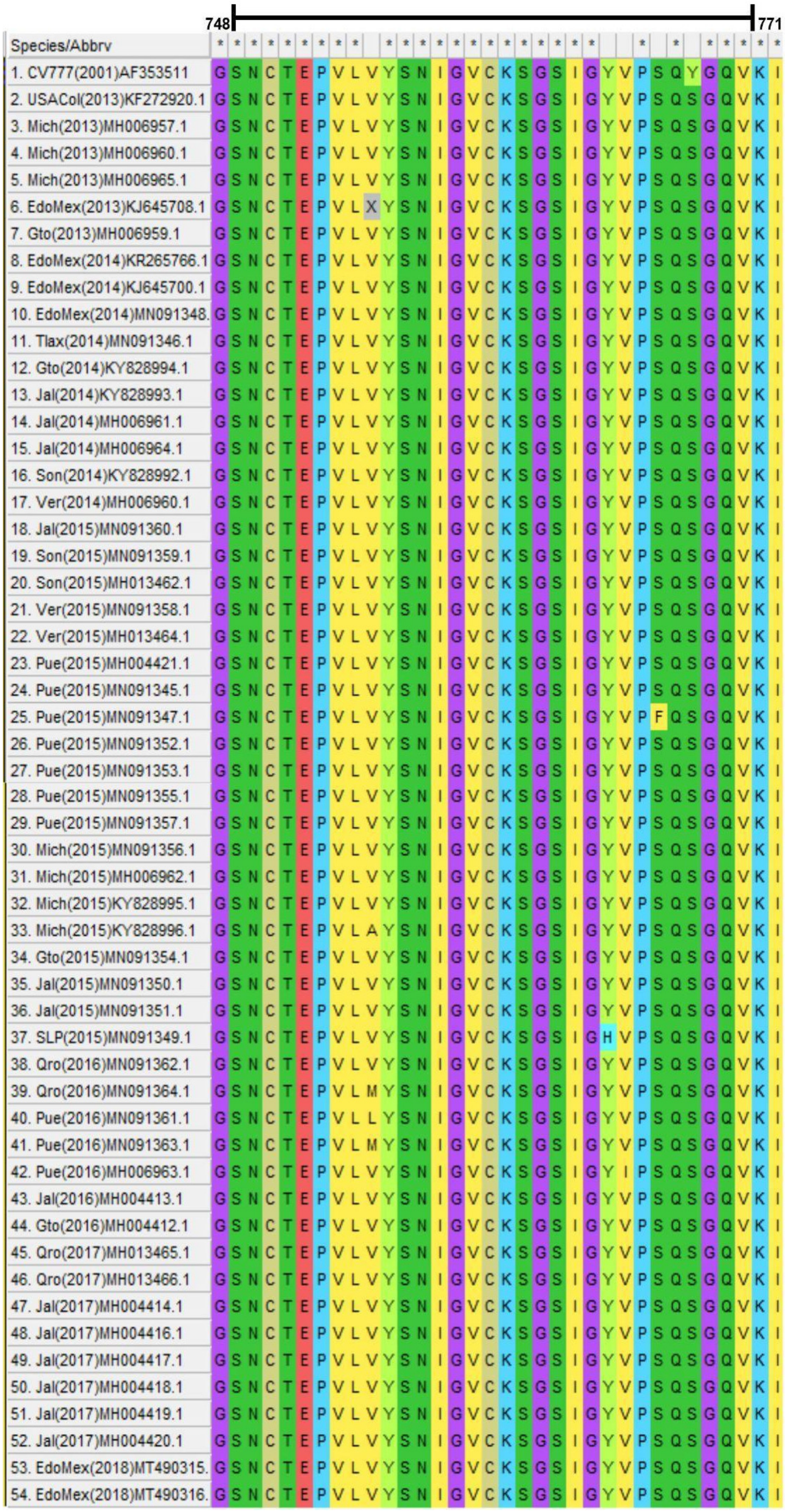

Figure S4. Alignment of amino acid residues of peptides derived from PEDV S glycoprotein. A) Amino acid alignment of the COE peptide derived from S glycoprotein of Mexican PEDV strains; B) Amino acid alignment of the S1D

peptide derived from S glycoprotein of Mexican PEDV strains; C) Amino acid alignment of the 2C10 peptide derived from S glycoprotein of Mexican PEDV strains; and D) Amino acid alignment of the SS2 and the SS6 peptides derived from S glycoprotein of Mexican PEDV strains. These data are part of YHG's thesis available at https://tesiunam.dgb.unam.mx/F/4TJIMMUAVBQNYPHGL8IYCHFAJM3EUNSIKHSCT1YKHUUTQA7VXT-05160?func=full-set-set&set_number=084027&set_entry=000001&format=999
