# Supplementary material for: Identification of Conserved B and T Cell Epitopes in Glycoprotein S of Mexican Porcine Epidemic Diarrhea Virus (PEDV) Strains via Immunoinformatics Analysis, Molecular Docking, and Immunofluorescence
Source: Viruses. 2026 Mar 25;18(4):407. doi: 10.3390/v18040407 (PMC13120105; doi:10.3390/v18040407)
Supplement: Supplementary file 1 [file viruses-18-00407-s001.zip › Figure S5 Alignment of B cell epitopes from PEDV S glycoprotein.pdf]

A) Amino acid alignment of the B cell epitopes

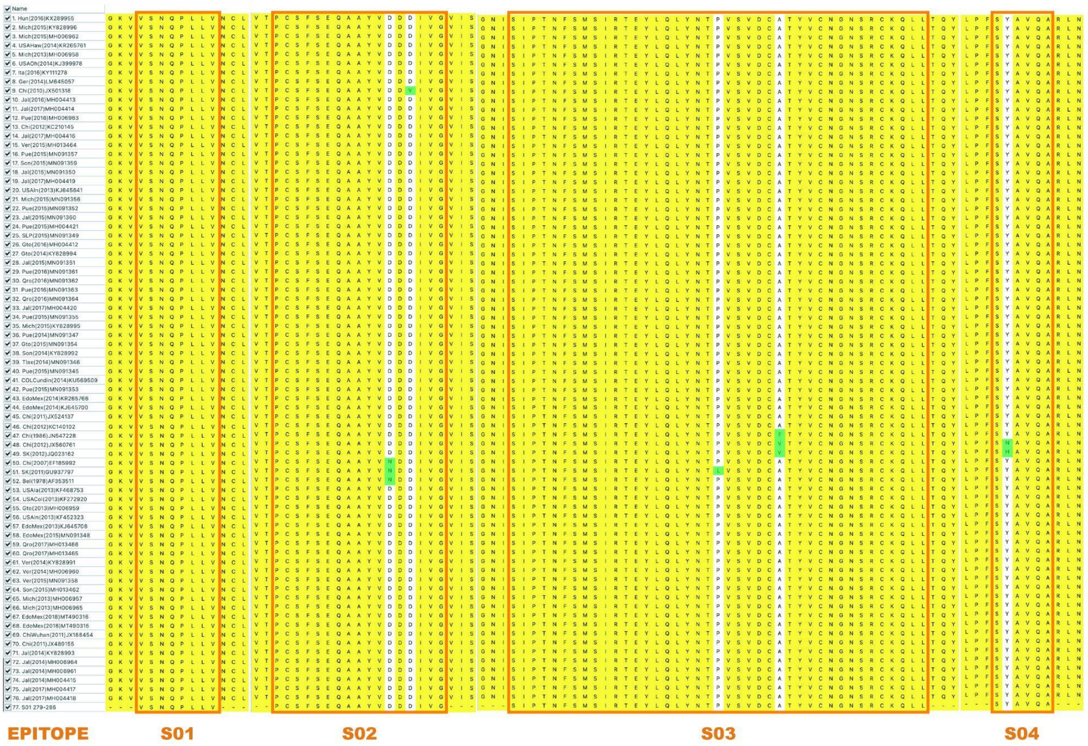

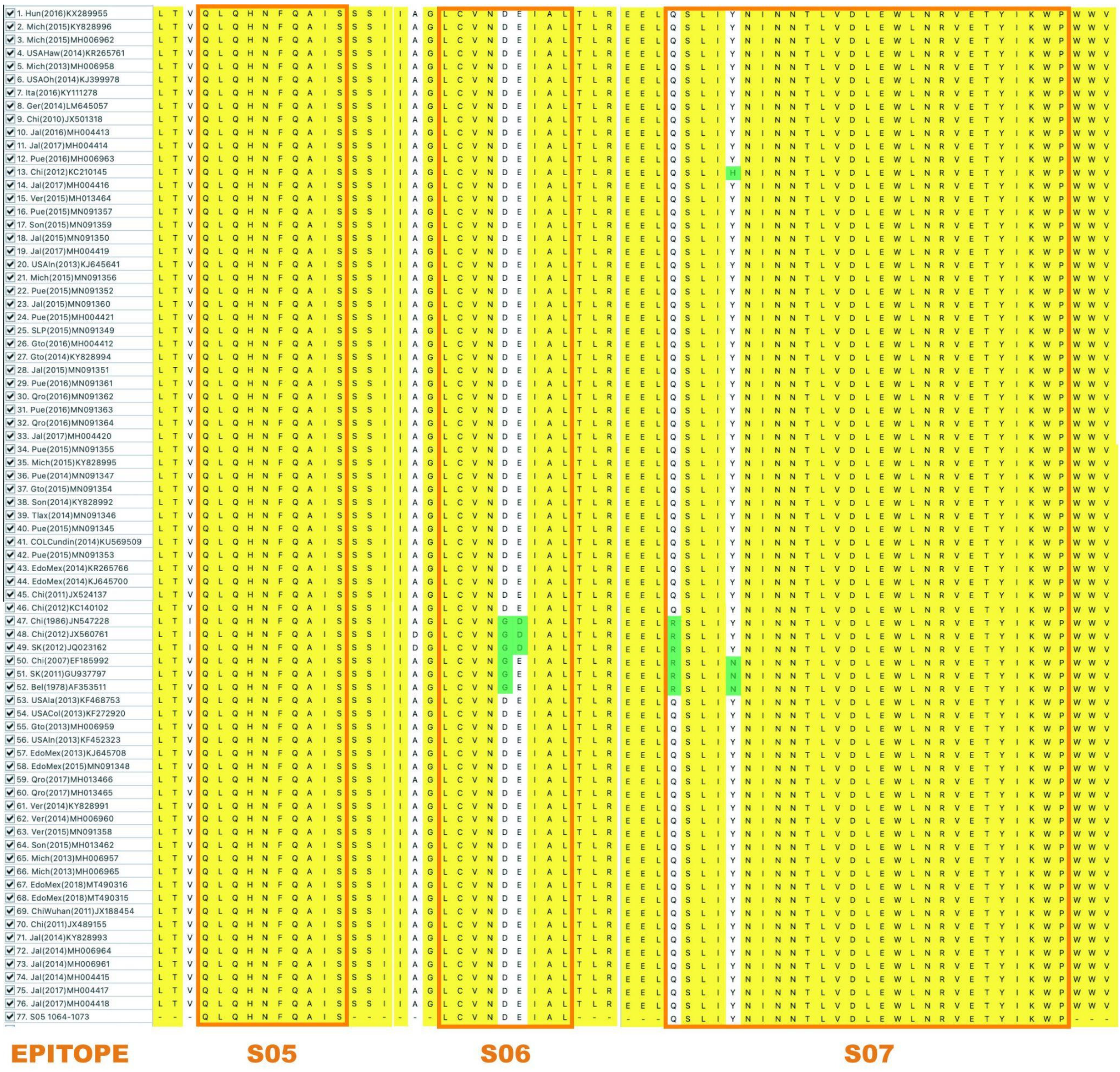
EPITOPE
S05
S06
S07

B) Amino acid alignment of HTL epitopes

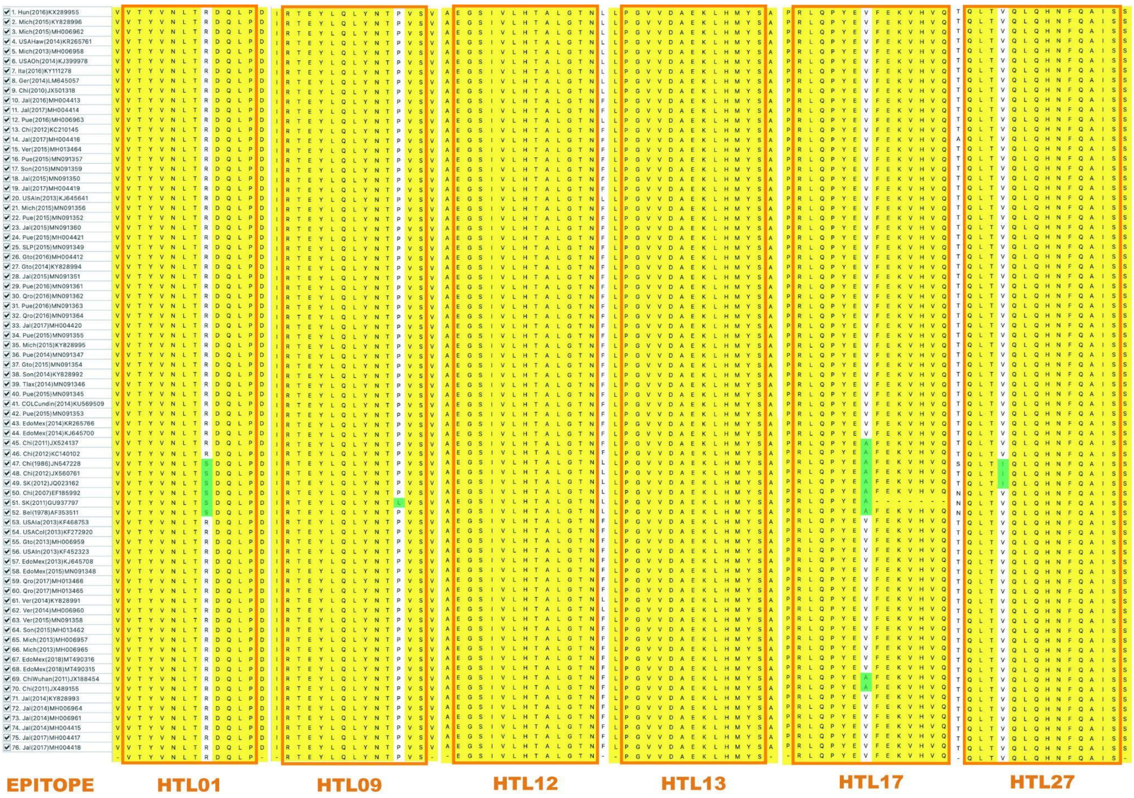

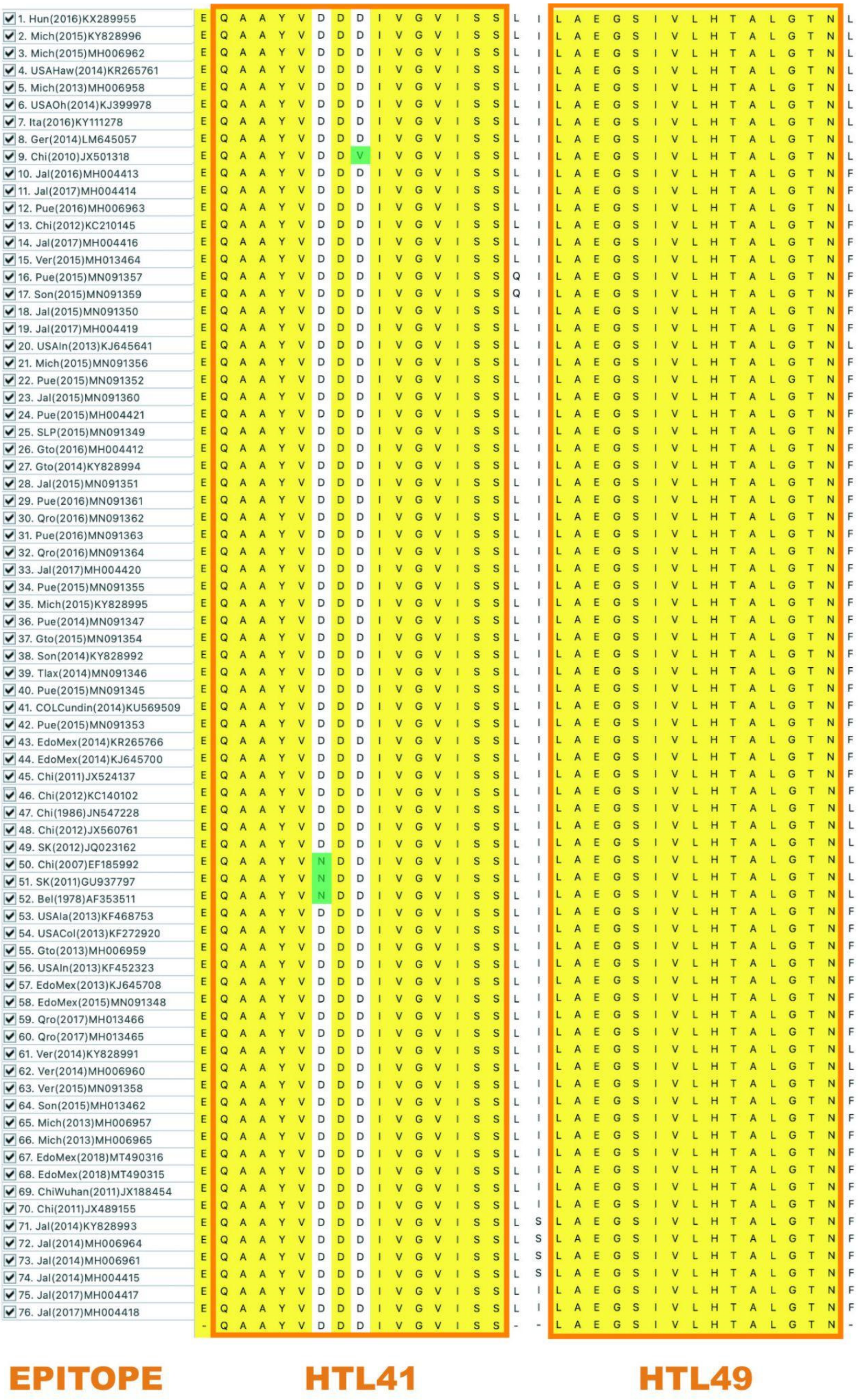
EPITOPE
HTL41
HTL49

C) Amino acid alignment of CTL epitopes

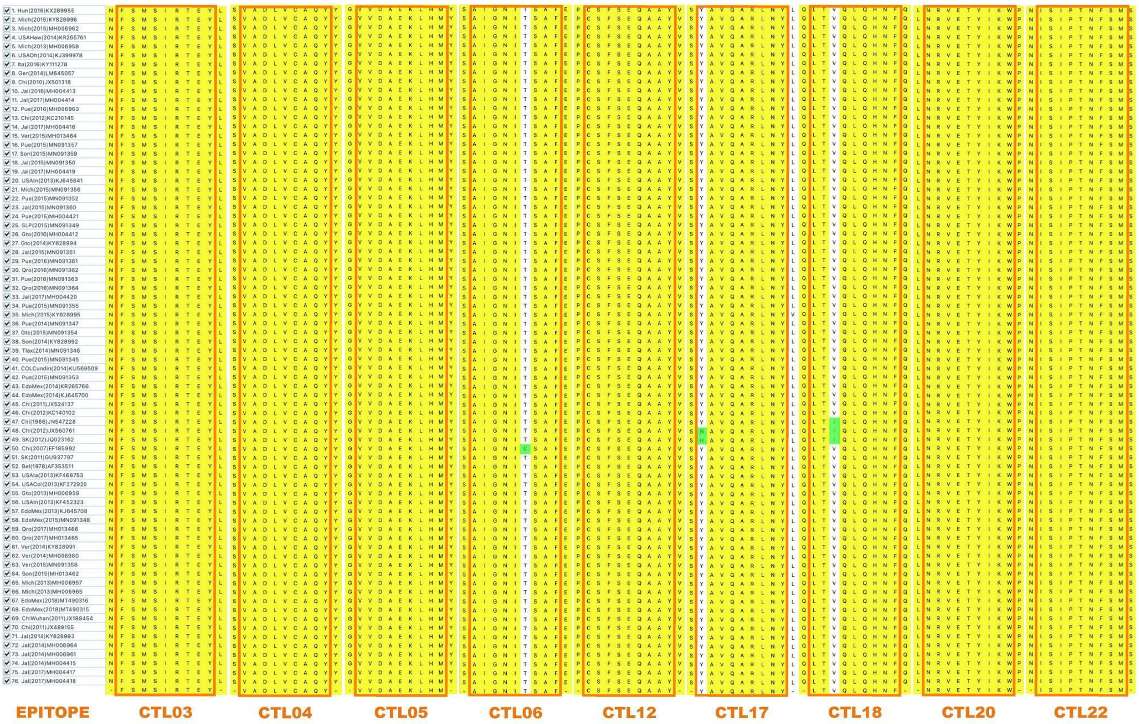

Figure S5. Alignment of amino acid residues of B, HTL and CTL epitopes derived from PEDV S glycoprotein among American, European and Asian PEDV strains. A) B cell epitopes; B) HTL epitopes; and C) CTL epitopes. Amino acid alignments were performed with muscle algorithm in Mega 11
